# A potential nomenclature for the Immuno Polymorphism Database (IPD) of chicken MHC genes: progress and problems

**DOI:** 10.1007/s00251-019-01145-6

**Published:** 2019-11-18

**Authors:** Hassnae Afrache, Clive A. Tregaskes, Jim Kaufman

**Affiliations:** 1 Department of Pathology, University of Cambridge, Tennis Court Road, Cambridge, CB2 1QP UK; 2 Department of Veterinary Medicine, University of Cambridge, Madingley Road, Cambridge, CB2 0ES UK

**Keywords:** BF-BL region, BF1, BF2, BLB1, BLB2, Recombination

## Abstract

Among the genes with the highest allelic polymorphism and sequence diversity are those encoding the classical class I and class II molecules of the major histocompatibility complex (MHC). Although many thousands of MHC sequences have been deposited in general sequence databases like GenBank, the availability of curated MHC sequences with agreed nomenclature has been enormously beneficial. Along with the Immuno Polymorphism Database-IMunoGeneTics/human leukocyte antigen (IPD-IMGT/HLA) database, a collection of databases for curated sequences of immune importance has been developed. A recent addition is an IPD-MHC database for chickens. For many years, the nomenclature system for chicken MHC genes has been based on a list of standard, presumed to be stable, haplotypes. However, these standard haplotypes give different names to identical sequences. Moreover, the discovery of new recombinants between haplotypes and a rapid increase in newly discovered alleles leaves the old system untenable. In this review, a new nomenclature is considered, for which alleles of different loci are given names based on the system used for other MHCs, and then haplotypes are named according to the alleles present. The new nomenclature system is trialled, first with standard haplotypes and then with validated sequences from the scientific literature. In the trial, some class II B sequences were found in both class II loci, presumably by gene conversion or inversion, so that identical sequences would receive different names. This situation prompts further suggestions to the new nomenclature system. In summary, there has been progress, but also problems, with the new IPD-MHC system for chickens.

**Electronic supplementary material:**

The online version of this article (10.1007/s00251-019-01145-6) contains supplementary material, which is available to authorized users.

## Introduction

The creation of an HLA Database of curated class I and class II nucleotide sequences marked a pivotal moment for the human major histocompatibility complex (MHC) community, beginning a process that allowed all researchers to use common and agreed names for particular well-characterised sequences (Zemmour and Parham 1991; Marsh and Bodmer 1991; Robinson et al. 2000). There have been many knock-on effects besides research into the MHC, such as providing the basis for single nucleotide polymorphisms (SNPs) to be used to impute MHC alleles for genome-wide association studies (GWAS), providing a model for other highly polymorphic immune gene systems such as the killer immunoglobulin-like receptors (KIRs), and providing the template for MHC systems in other species (Maccari et al. 2018; Robinson et al. 2015).

In addition to the database of human MHC sequences, now called Immuno Polymorphism Database-IMunoGeneTics/human leukocyte antigen (IPD-IMGT/HLA), the IPD hosts a variety of databases, each of which has expert curators to ensure that the sequences are validated and named according to appropriate nomenclature. Among these databases are those including MHC sequences (IPD-MHC) from non-human primates (NHP), bovids (including cattle), caprids (including goats), canids (including dogs), equids (including horses), murids (including rats), ovids (including sheep), suids (including swine), salmonids (including fish) and avians (including chickens) (Ballingall et al. 2018, Maccari et al. 2017, 2018). This review describes the progress made and the problems encountered in assembling, curating and extending the alleles of classical class I and class II B genes from the chicken MHC in order to properly implement the chicken database for IPD-MHC, efforts that continue to require serious reconsideration of the genetic nomenclature that has been in place for decades.

## Discovery and first analyses of the chicken MHC

After the discovery of the mouse H-2 complex but before reports that led to the human HLA complex, a series of antigenic systems for chicken blood cells were described by Edwin Briles and co-workers (Briles et al. 1950). These systems were discovered by injection of whole blood or blood cells from one chicken to another followed by haemagglutination to detect antibodies. As part of the effort to understand these serologically detected systems, lines of chickens were bred to be homozygous for antigenic alleles of blood group B (Abplanalp et al. 1981; Gilmour 1959), and functional assays emblematic of the MHC, including graft rejection, mixed lymphocyte reaction, graft-versus-host reaction, immune response to limited epitopes and autoimmunity, were found to be determined by the B locus (Bacon et al. 1973; Gebriel and Nordskog 1983; Schierman and Nordskog 1961, 1963; Vilhelmova et al. 1977).

Comparison of the patterns of serological reaction from different lines of chickens, along with absorbing populations of antibodies with various cell types from different lines, divided the B locus into a BG region that determined polymorphic erythrocyte antigens, and a BF-BL region that determined BL antigens found on lymphocytes and BF antigens on both erythrocytes and lymphocytes (Simonsen et al. 1982). By immunoprecipitation and gel electrophoresis of radiolabelled molecules, the BF and BL antigens were found to the equivalent of class I and class II molecules respectively, while the BG antigens (also by this time called class IV antigens) were something else entirely (Wolf et al. 1984). Interest in these regions was heightened by strong associations with economically important diseases such as Marek’s disease, for which particular B locus alleles, eventually located in the BF-BL region, conferred striking resistance or susceptibility (Briles et al. 1977, 1983; Plachy et al. 1992; reviewed in Miller and Taylor 2016).

**Fig. 1:**
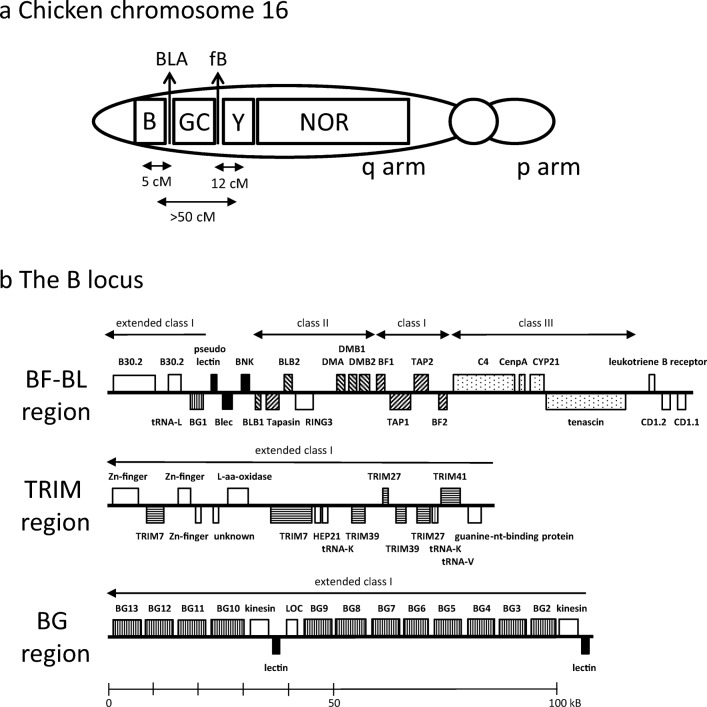
Organization of regions on chicken chromosome 16, as currently understood. **a** Depiction of chromosome 16, based on analysis by FISH, radiation hybrids, genetics, southern blotting and sequencing. **b** B locus; GC, G + C rich region; Y, Rfp-Y region; NOR, nucleolar organiser region; BLA, class II A gene; fB, factor B gene. Double-headed arrows indicate recombination frequencies for between B and BLA, fB and Rfp-Y and B and Rfp-Y. B. Region of the B locus currently sequenced, including the BF-BL region, the TRIM region and the BG region. Genes are represented by boxes. Rising and falling stripes indicate genes of the classical class I and class II presentation system, respectively; stippled indicate class III region genes; black indicates lectin-like genes and pseudogenes; horizontal stripes indicate TRIM family genes; vertical stripes indicate BG genes. Names of genes above indicate transcription from left to right, below indicate transcription from right to left. References to support sequence data and identifications in Kaufman 2013, from which this figure and figure legend are taken (with permission), except for the BG region (Salomonsen et al. 2014)

The B locus was found with the ribosomal RNA genes of the nucleolar organiser region (NOR) on chicken chromosome 16 (Bloom and Bacon 1985), a microchromosome that is still in the process of being completely sequenced. After the seminal description of cosmid clones bearing class I and class II B genes (Guillemot et al. 1988) followed by many studies utilising molecular biology, our current understanding (Fig. 1a) has the B locus containing the classical MHC on the telomeric side of the long arm of this chromosome, followed by a region of repeats and the so-called Rfp-Y region containing non-classical class I and class II genes, then the NOR followed by regions containing scavenger receptor and olfactory receptor genes (reviewed in Kaufman 2013).

The B locus is now understood to include a multigene family of BG genes in the BG region, then a region containing many TRIM among other genes, then the BF-BL region containing the classical MHC genes, and at the end of the sequenced region a pair of CD1 genes, non-polymorphic non-classical class I genes that in mammals are located in an MHC-paralogous region on a different chromosome (Fig 1b). Within the BF-BL region are the polymorphic classical class I and class II B genes (Hosomichi et al. 2008; Hunt and Fulton 1998; Jacob et al. 2000; Kaufman et al. 1999; Shaw et al. 2007; Pharr et al. 1998; Wallny et al. 2006), along with the genes involved in antigen processing and peptide loading: DMA, DMB1, DMB2, tapasin, TAP1 and TAP2, all of which are also polymorphic (Atkinson et al 2001; Chazara et al. 2011; Hosomichi et al. 2008; van Hateren et al. 2013; Walker et al. 2005, 2011). In addition, several other genes are located in the BF-BL region: a BG gene (BG1), a natural killer (NK) receptor gene and a potential ligand (BNK and Blec), the transcription factor RING3 (Brd2, found in every MHC carefully examined), the complement component C4, a structural gene tenascin and the enzyme steroid hydroxylase. BG1 and BNK are known to be highly polymorphic (Chattaway et al. 2016, Hosomichi et al. 2008, Rogers and Kaufman 2008).

## The first nomenclatures of the B locus

Nomenclature to describe B alleles began with the serological definition of standard haplotypes, starting with B1 (Briles et al. 1982). As the complexity of the B locus was better appreciated and the molecular definition of genes and alleles progressed, this system was retained but with a variety of gene names followed by allele numbers. The currently published system of nomenclature (Miller et al. 2004) recognises 29 haplotypes and utilises gene names based loosely on the original description from the cosmids of the B12 haplotype from the CB congenic chicken line along with later sequencing of the B locus cosmids (Guillemot et al. 1988, Kaufman et al. 1999), so the polymorphic class II B genes are called BLB1 and BLB2, and the polymorphic class I genes are called BF1 and BF2 (Fig. 1b). In addition, non-classical class I genes from the Rfp-Y region were found among the original cosmids and to be polymorphic (Afanassieff et al. 2001; Miller et al. 1994; Zoorob et al. 1993), so these YF genes were also incorporated into the published nomenclature system. The gene names are separated from the allele numbers by a star (or asterisk), with allele names coming from the surviving 20 standard B haplotypes but organised with allele groups in the first field separated by a colon from closely related variants in the second field, based on the system originally developed for human MHC genes and then extended to other vertebrates (as currently described with some modifications in Ballingall et al. 2018). Thus, the BF2 gene of the B2 haplotype (and for which only one sequence variant was described) has been named BF2*02:01. Many such sequence alleles are listed along with GenBank accession numbers and the chicken lines in which they were found (Miller et al. 2004).

This currently accepted nomenclature system has been in place for 15 years, but there have been some difficulties in application. First, not all the sequences are known (Miller et al. 2004). It would appear that B25 through B29 have not been analysed at the molecular level. More seriously, the chicken lines for some standard haplotypes have apparently been lost without the sequences of their alleles being known (Miller et al. 2004). So, the molecular identities of B1, B3, B10, B16, B20, B25, B27, B28 and B29 may never be known.

Second, the BLB and BF genes are apparently identical in some existing standard haplotypes. So, B4 and B13 differ in the BG region (the predominant determinant of the original serological identity) but are nearly identical in the sequenced genes of the BF-BL region (Hosomichi et al. 2008; Hunt and Fulton 1998; Pharr et al. 1998; Jacob et al. 2000; Shaw et al. 2007; Walker et al. 2005; Wallny et al. 2006).

Third, some published gene sequences for lines considered to have the same B haplotype are not the same, potentially due to issues of breeding, or to nucleotide mis-incorporation during PCR or within the bacteria during cloning. For example, a sequence for a clone representing the dominantly expressed class I gene from a B19 homozygote line of chickens was reported, followed later by a publication re-naming this sequence as B19var1, on the grounds that the “B19 type line” was held in a different institution and had a different sequence (Hunt et al. 1994; Kaufman et al. 1992). The second publication in fact used a congenic line derived from the type line, and subsequent analysis showed that the original sequence was correct (Hosomichi et al. 2008), but not before the B19 and B19var1 names were used widely in the scientific literature (including Miller et al. 2004). Similarly, some older sequences from clones derived from well-known lines do not agree with current sequences and may be due to inclusion of primer sequences during PCR or to nucleotide mis-incorporation during cloning (for example, Hunt and Fulton 1998; Liu et al. 2002, Wallny et al. 2006)

Fourth, recombination while rare does occur within the BF-BL region. The first example identified was the standard B19 haplotype, which by serology had BL in common with the B12 haplotype and BF closely related to the B15 haplotype (Simonsen et al. 1982). It is now clear by genomic sequences that the B19 haplotype is a hybrid of the B12 and B15 haplotypes, with the recombination site in the middle of the TAP gene and with a few nucleotide changes acquired in some genes (Hosomichi et al. 2008; Jacob et al. 2000; Rogers and Kaufman 2008; Shaw et al. 2007; van Hateren et al. 2013; Walker et al. 2005, 2011; Wallny et al. 2006). Several other examples among the standard haplotypes have since been identified, including recombination events apparently giving rise to the B5, B8 and B11 haplotypes found in various chicken flocks (Hosomichi et al. 2008).

Fifth, comparison of genomic sequences for many BF-BL haplotypes identified long stretches of identical sequence between two haplotypes with completely different flanking sequences (Hosomichi et al. 2008). These results were interpreted as “gene conversion”, although in the absence of evidence about the homologous chromosome during meiosis, these could easily be due to “double reciprocal recombination” events rather than gene conversion as originally defined (Suyama et al. 1959; Baltimore 1981). In any case, it has become clear that more complex events than just simple single recombination take place in the BF-BL region.

Finally, many sequences have been deposited in general sequence databases (like GenBank) that are not the same as the sequences known from the 29 standard haplotypes. Virtually all of these sequences come from PCR and only some have been controlled (typically by comparing results of independent PCRs) for nucleotide mis-incorporation or chimerism; only a few have been reported in publications. Moreover, there has been no clear mechanism to decide additional haplotype or allele numbers.

Of all these difficulties, only the nomenclature of recombinant haplotypes has been approached in the current system (Miller et al. 2004). It was decided that the allele number of the BF2 sequence (rather than the serology primarily of the BG region) would be used to name the B locus haplotype, with recombinants given the designation “r” followed by a number in order of discovery. Thus, B2r1 would be the first recombinant described between the BF2 gene of the B2 haplotype but without information about the rest of the haplotype.

## A potential new nomenclature for chicken MHC alleles and haplotypes

The question of a new nomenclature arose when considering how to curate allelic sequences for the chicken section of the IPD-MHC. It seemed likely that there would be many more sequences and haplotypes than the standard haplotypes examined up to now, and there was ample evidence for simple recombination and more complex events in the BF-BL region. Therefore, the current system of haplotypes followed by recombination numbers no longer seemed tenable, and a more descriptive and flexible system based on gene sequences was considered desirable.

The current form used for naming chicken MHC genes follows the system developed for humans and extended to other vertebrate groups (Ballingall et al. 2018; Maccari et al. 2017, 2018; Miller et al. 2004). As mentioned above, the gene name is followed by a star (asterisk), then the number of the allele group (also referred to as a “designation”) is followed by a colon (often referred to as the “first field”), and then the number of a non-synonymous variant within the allele group is followed by a colon (the second field). In the IPD nomenclature, a third field is the number of the synonymous variant within the variant group followed by a colon, and a fourth field gives the number of the variant in non-coding regions.

However, radical changes in the chicken nomenclature are envisaged for the naming of the allele groups and the definition of haplotypes. Instead of all genes within a defined haplotype having the number of that haplotype without regard to sequence, each gene (genetic locus) would have a list of sequence alleles and a haplotype would be named after the sequence alleles present in that haplotype. Thus, a particular sequence would have a single allele name regardless of haplotype. Also, closely related sequences would have the same first field but differ in the second and/or third fields (and as more complete data is obtained, in the fourth fields), rather than reflect a haplotype number. These potential changes bring the chicken nomenclature more into line with the recommendations for IPD databases (Ballingall et al. 2018; Maccari et al. 2017, 2018).

In deference to the decades of extensive and outstanding research into the chicken MHC, it seemed appropriate to treat the sequences in three groups: the sequences of the standard haplotypes for which the most information is available, the sequences outside of the standard haplotypes that are published with suitable controls and replication, and the sequences that are only found in existing general sequence databases or are currently being identified.

For the existing standard haplotypes, the process would be to compare the sequences of each genetic locus between all haplotypes, then start with the lowest numbered haplotype to name identical and closely related sequences appropriately with allele and variant names, then consider the haplotype with the next lowest number and so forth, naming any unique sequences with the allele number of that haplotype. Thus, in the absence of an available B1 haplotype, the sequences found in the B2 haplotype would be given the names BLB1*002:01:01, BLB2*002:01:01, BF1*002:01:01 and BF2*002:01:01.

For the sequences in the literature that are outside the standard haplotypes, almost all have entries in general sequence databases, some are described to be in haplotypes, and almost all are partial genes amplified by PCR from genomic DNA. Most usually, exon 2 from BLB genes and exons 2, 3 or both from BF genes are reported, but occasionally further exon or intron sequence is available; only those BF sequences that included both exon 2 and exon 3 would be considered. The first step would be to ensure that independent amplifications have given the same results; if not, the sequence would not be considered valid. As a second step with validated sequences, the gene from which the sequence was likely to have been derived would be inferred, then this sequence would be compared to the alleles of that genetic locus from the standard haplotypes, identical or closely related sequences are named accordingly, and finally any unique sequences would be given allele numbers starting with 30, well above the standard haplotypes for which sequences are available.

For those sequences that are only found in standard sequence databases such as GenBank (as well as those that are being discovered in an MHC typing effort described below), the first requirement would replication, validating only sequences found from independent amplifications (from independent experiments with the same bird, or from different birds, lines or studies). Sadly, most sequences in GenBank are present only once, and often the sequences in different entries from the same study differ only in one or two nucleotides and are most likely to be the result of nucleotide mis-incorporation during PCR for which no controls have been performed (Online Resource 1). Those sequences with replication would be processed in the same way as the sequences from the literature.

## Application of the potential new nomenclature to the standard B haplotypes

To carry out the process described above for the standard B haplotypes, two analyses based on alignments of both nucleotide and amino acid sequences were employed: phylogenetic trees to establish apparent clades and distance matrices to establish the number of sequence differences. The alignments were first carried out for exon 2 to exon 3 of the classical class I sequences (the peptide-binding domain without the intervening intron, which is anyway nearly invariant, Shaw et al. 2007), and for exon 2 of the classical class II B sequences (without the rest of the gene, which is also nearly invariant, Jacob et al. 2000), and later extended to the whole coding sequence (CDS equivalent) when possible. These alignments are relatively straightforward since there are no indels in the exons encoding the peptide-binding domains between any of the class I sequences or between any of the class II B sequences.

However, one might argue that a more sensible approach would be to compare alleles from a particular locus, rather than all class I sequences and all class II B sequences, given that differences in function might lead to differences in the sequence positions of variation. The biological functions of the polymorphic classical MHC loci, based on expression level and tissue distribution primarily from cDNA along with functional data mostly from in vitro cellular immunology, suggest that there is good reason to consider the alleles of these loci separately. Both class I loci are widely expressed, but the BF2 gene is strongly expressed and recognised by cytotoxic T lymphocytes (CTLs), while the BF1 gene is relatively poorly expressed and recognised by NK cells (Ewald and Livant 2004; Fulton et al. 1995; Kim et al. 2018; Juul-Madsen et al. 2000; Shaw et al. 2007; Thacker et al. 1995; Wallny et al. 2006). In contrast, the class II genes differ in their tissue distribution: the BLB2 gene is strongly expressed in many tissues, but the BLB1 gene is poorly expressed except in the intestine (Jacob et al. 2000; Parker and Kaufman 2017; Pharr et al. 1998). The assignment of particular sequences to the polymorphic classical MHC loci was possible for many of the standard haplotypes, based on complete BF-BL region sequences (Hosomichi et al. 2008; Kaufman et al. 1999) along with supporting data from complete or partial cDNA and gene sequences available from one or more chicken lines (Online Resource 2), so lists of alleles were established for each genetic locus separately.

**Fig. 2:**
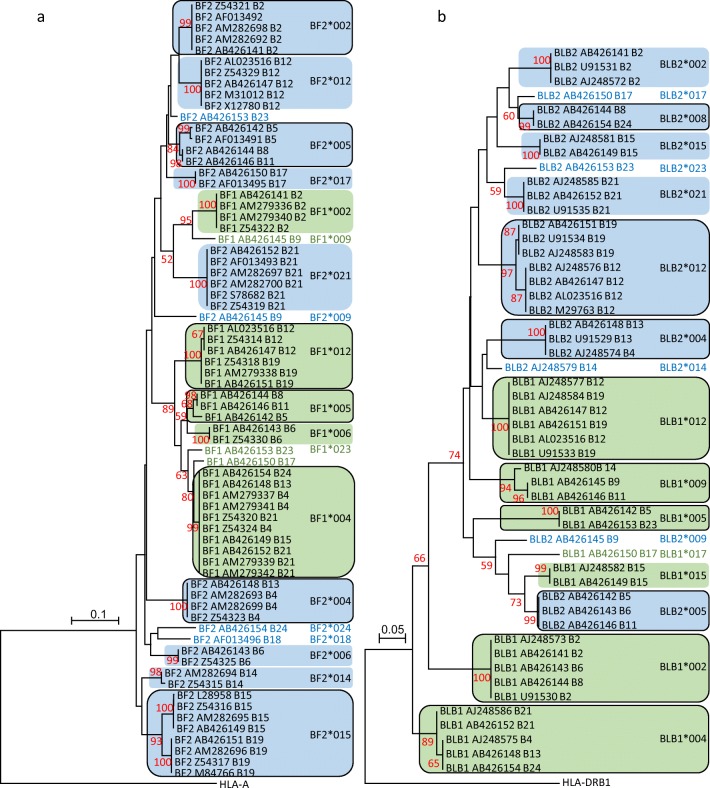
Phylogenetic trees for amino acid sequences of MHC peptide-binding domains from standard haplotypes. **a** α1 and α2 domains of BF sequences (with the first and last seven amino acids removed, corresponding to primers and other reasons for different lengths of sequence); **b** β1 domains of BLB sequences (with the first two amino acids removed, corresponding to primers and other reasons for different lengths of sequence). Neighbour joining (NJ) trees were created by MEGA7 (Kumar et al. 2016) using the Jones-Taylor-Thornton (JTT) matrix-based method (Jones et al. 1992) using sequences from the GenBank accession numbers on the tree and with human sequences as outgroups. Genetic distances are indicated with bars; red numbers are bootstrap values (percentages) for those nodes that reach significance from 500 replications; names at the tips are the gene name, followed by the GenBank accession number, followed by the haplotype. Allele groups for BF1 and BLB1 (or BF2 and BLB2) are named, either in green (or blue) for single sequences or in black surrounded by green (or blue) background for clades with more than one sequence; the coloured background for clades with sequences from more than one haplotype are surrounded by a black line

To establish apparent clades that could be considered allele groups of variants for the first and second fields of the name, phylogenetic trees were used. Both nucleotide and amino acid sequences were used, of which examples for the amino acids from the α1 and α2 domains (and nucleotides for exons 2–3) of BF and from the β1 domains (exon 2) of BLB sequences are shown (Fig. 2; Online Resource 3).

Typically all the BF sequences from a particular B haplotype are identical, but not necessarily all BF sequences for a particular clade (Fig. 2a; Online Resource 3a). For instance, the five BF2 amino acid sequences from the B2 haplotype in the top clade are identical despite originating from multiple chicken lines and being analysed by different researchers in different ways; the same is true for the nucleotide sequences. Similarly, all the BF2 sequences from each of the B6, B12, B17 and B21 haplotypes and the BF1 sequences from each of the B2 and B6 haplotypes are identical within their clades, both as amino acids and as nucleotides. The two BF2 sequences from the B14 haplotype differ in one nucleotide leading to one amino acid difference; this appears to be due to a cloning artefact. However, other clades have sequences from more than one B haplotype. For instance, the BF2 sequences from the B4 and B13 haplotypes are identical in exons 2 and 3 at the amino acid and nucleotide levels. Similarly, BF2 sequences from the B15 haplotype are identical as are those from the B19 haplotype, but together they form a clade of closely related sequences. The same is true for both the BF2 sequences of the B5, B8 and B11 haplotypes. Very striking is the BF1 clade of B4, B13, B15, B21 and B24 sequences, which are identical in these exons, although they can differ slightly in other exons. Only single sequences are available for BF1 from the B9, B17 and B23 haplotypes, as well as BF2 from the B9, B18, B23 and B24 haplotypes. In fact, the BF1 sequence from the B17 haplotype appears on its own just outside the B4 clade, but was considered to be part of the B4 allelic group based on distances matrices, as discussed below. Similarly, the BF2 sequences from the B2 and B23 haplotypes seem to be in different clades for amino acids, but arguably in the same clade for nucleotides; the decision to consider them as the same allelic group was based on distance matrices.

Overall, there are fewer clades for BF1 than BF2 sequences, and there are no BF1 sequences clustered in the same narrow clade with BF2 sequences in this tree. However, in some analyses not shown here, the BF sequences form two much larger clades: most BF1 sequences in one large clade and all the BF2 sequences along with the BF1 sequences from the B2 and B9 haplotypes in the other large clade. Thus, on the basis of sequence alone, there would be a reasonable probability of assigning a newly discovered BF sequence that is not closely related to an existing sequence to the right genetic locus.

The BLB sequences overall show similar features, except that BLB1 and BLB2 sequences are more intermixed (Fig. 2b; Online Resource 3b). For haplotypes with multiple sequences in the literature, they are identical for each haplotype, but only BLB1 from B15 and BLB2 from B2, B15 and B21 each form their own clades. Some clades are only represented by a single sequence: BLB1 from B17, and BLB2 from B9, B14, B17 and B23. All other sequences are in clades with several haplotypes. Clusters of BLB1 sequences are found for B2, B6 and B8; for B4, B13, B21 and B24; for B5 and B23; for B9, B11 and B14; and for B12 and B19. Clusters of BLB2 sequences are found for B4 and B13; for B5, B6 and B11; for B8 and B24; and for B12 and B19. There are no instances of BLB1 and BLB2 sequences being closely related in a well-supported clade, but several examples of more distantly related BLB1 and BLB2 sequences together in moderately supported clades. Thus, on the basis of sequence alone, it may become problematic to assign a newly discovered BLB sequence to the right genetic locus, unless it is very closely related to an existing sequence. In fact, this possibility turned out to be even more of a problem than anticipated, as described in more detail below.

**Fig. 3:**
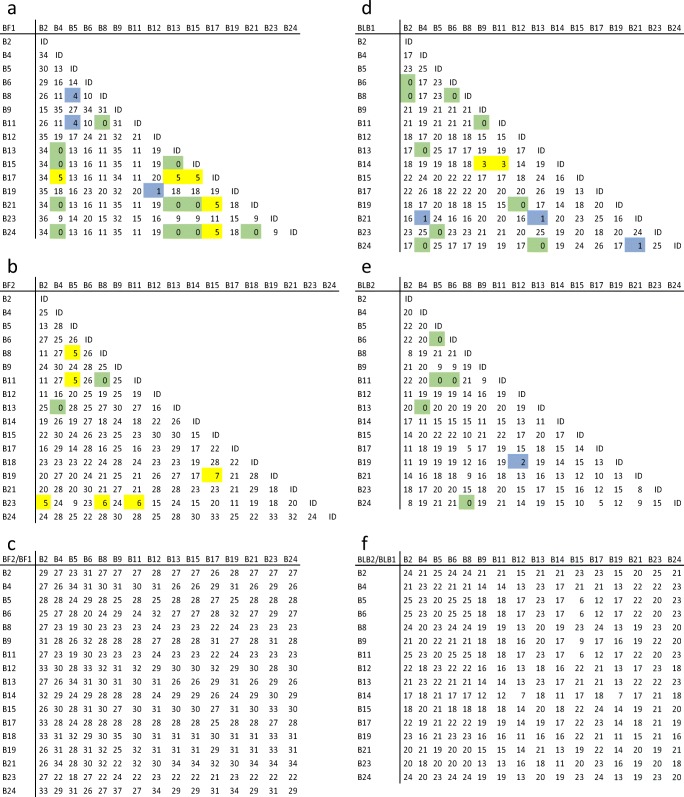
Distance matrices for amino acid sequences of MHC peptide-binding domains from standard haplotypes, with the α1 and α2 domains of **a** BF1 versus BF1 alleles, **b** BF2 versus BF2 alleles, **c** BF1 versus BF2 alleles, and with β1 domains of **d** BLB1 versus BLB1 alleles, **e** BLB2 versus BLB2 alleles, **f** BLB1 versus BLB2 alleles. The sequences used are the consensus full-length domains, without truncation. Alignments were performed using MAFFT online (Katoh et al. 2002; https://mafft.cbrc.jp/alignment/server/) and the results were pasted into Bioedit (Hall 1999; https://softfamous.com/bioedit/) on a desktop computer; the command “Sequence difference count Matrix” under “Alignment” was used to generate the distance matrix, which was pasted into Microsoft Excel and then Powerpoint for producing the final figure. Highlights indicate differences for BF (or BLB): green, none; blue, 1 to 4 (1 or 2); yellow, 5 to 8 (3 or 4); ID, comparison between the same sequence

Once the allele groups were established based on clades, they were analysed by distance matrices, starting with amino acids (Fig. 3) and then nucleotides (Online Resource 4). Initially, only exon 2 (β1 domain) of BLB sequences, and exons 2 and 3 (α1 and α2 domains) of BF sequences were compared, given that these regions determine the peptide-binding specificities. However, there are at least three concerns with this approach, which were rationalised as follows. First, not all the polymorphic positions within these exons/domains contribute to peptide-binding, but it was judged that the number of variable positions outside of the peptide-binding groove was likely to be small and therefore not affect assignments too much. Second, some variation in the whole gene/molecule can be found outside of these peptide-binding exons/domains, but it was judged that many of the available sequences outside of the standard haplotypes would not have more sequence than these exons/domains, and after the initial characterisation further adjustments based on the few additional differences could be made. Third, the relationships of recombinant alleles (for instance, a BF gene composed of exon 2 from one BF allele and exon 3 from another BF allele) would not be captured, but it was judged that there was no simple way to present this information in a name and that further characterisation might compare BF exon 2/α1 and exon 3/α2 sequences separately to look for interesting differences. In any case, after the initial examination, the analyses were extended to the whole protein (Online Resource 5).

Overall, it turned out to be relatively easy to make sensible assignments for the standard haplotypes, based on these difference matrices. Most of the BF sequences differed by over 20 amino acids, while most of the BLB sequences differed by at least 12 amino acids. A few sequences for each were identical (green highlights in Fig. 3), and a few more had only one or two amino acid differences per domain (blue highlights). The difficult judgement was how many more differences were too many to be a variant within an allele group. As for human alleles, four amino acid differences per domain (totalled to eight for BF sequences) were used as a cut-off (yellow highlights). This felt to be reasonable since the BF2 molecule from the B19 haplotype is known to be derived from the B15 haplotype, and differs in seven amino acids over the α1 and α2 domains. Those comparisons with more than five but less than ten differences per exon were considered not to be variants of each other, but to warrant further analysis in the future.

**Fig. 4:**
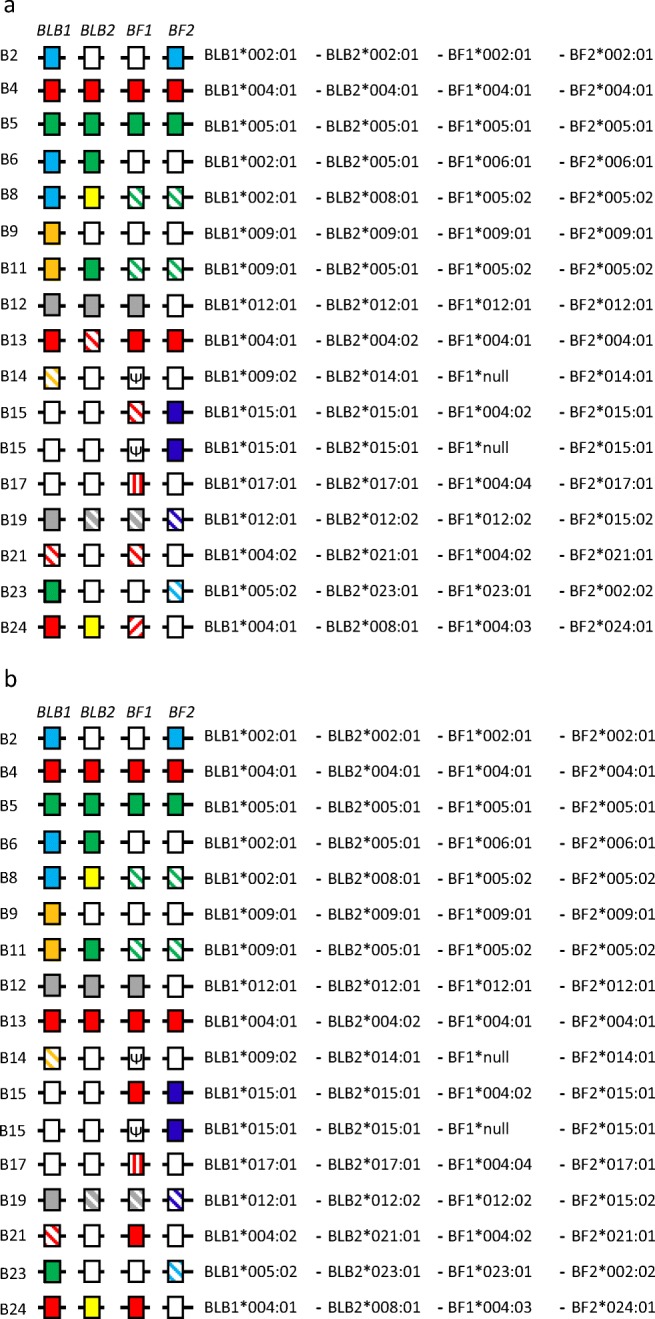
Haplotypes with genes as boxes for the standard haplotypes, with haplotype strings of the potential allele names. Comparisons based on **a** full coding sequences (CDS), **b** peptide-binding domains/exons. Unique sequences for each allelic series are indicated as white boxes, identical sequences are the same colours and closely related variants have the same colours but are striped in different ways for different variants of the same allele group. Note: the boxes for some alleles that are striped as variants considering the whole CDS in Fig. 4a are not striped in Fig. 4b since the there are no differences in the peptide-binding domains/exons. GenBank accession numbers and citations for the sequences are in the legend of Online Resource 2

Once the alleles and variants were established, then they were named in numerical order of haplotype. Illustrations of those decisions for the standard haplotypes are given for the whole amino acid coding sequence (CDS) (Fig. 4a) and for the peptide-binding domains (Fig. 4b), in which unique sequences have no colour, identical sequences are coloured and close variants have the same colour but are striped. Given that there are no sequences available for the B1 haplotype (Miller et al. 2004) and the type line is no longer in existence (S. Lamont, personal communication), the first standard haplotype known to be available is B2, for which there are sequences from multiple lines and sources. Thus, all the genes would receive the same allele number reflecting that basal haplotype, for example BLB1*002:01:01. There are again no B3 sequences or chicken lines available, but the sequences for B4 are not similar to B2, and the sequences for B5 are not similar to either B2 or B4. Thus, the allele numbers would reflect these haplotypes, for example BLB1*004:01:01 and BLB1*005:01:01.

The situation becomes more complicated for haplotypes with higher B numbers (Fig. 4a, b). For the B6 haplotype, the BF1 and BF2 genes are different from B2, B4 and B5, but the BLB1 sequence is identical to B2 throughout the coding sequence and is thus named BLB1*002:01:01, while the BLB2 sequence is identical to B5 throughout the coding sequence and is thus named BLB2*005:01:01. For the B8 haplotype, BLB1 is identical to B2 (and also B6) and so it would be named BLB1*002:01:01, BLB2 is not like any of the haplotypes with a lower number (but is identical to BLB2 from B24) and would be named BLB2*008:01:01, and both BF1 and BF2 are amino acid variants of the B5 genes (and are identical to the B11 genes) and would be named BF1*005:02:01 and BF2*005:02:01. The rest of the haplotypes for which there are sequences available would be named in a similar way, except that the B14 and one B15 haplotype have null alleles for BF1, while another B15 haplotype has BF1*004:02:01. All four genes of the B13 haplotype are identical to B4 in the peptide-binding region (although the BLB2 sequence for B13 differs in one nucleotide leading to one amino acid change in exon 3), so they all would have names that reflect this fact, giving the haplotype BLB1*004:01:01-BLB2*004:02:01-BF1*004:01:01-BF2*004:01:01. Also, the BF2 gene from the B19 haplotype is extremely similar to the B15 haplotype, and the other three genes are identical or very similar to the B12 haplotype, so this haplotype becomes BLB1*012:01:01-BLB2*012:02:01-BF1*012:02:01-BF2*015:02:01.

Overall, it can be seen that most haplotypes are patchworks of identical or similar sequences shared with other haplotypes, suggesting recombination or other processes over a considerable period of time. Moreover, some allelic groups have many variants, particularly obvious for the BF1*004 clade, although there are additional differences outside of exons 2 and 3 for some variants (comparing Fig. 4a with Fig. 4b). This is not a drawback for the standard haplotypes, for which there are complete gene sequences for all alleles, but becomes a difficulty below when comparing sequences from the literature, for which typically only partial sequences are available.

A serious drawback to this new nomenclature is that it is quite cumbersome. A convenient shorthand for the complicated gene name might be to leave out the last fields that are identical to the first variant described (so that BF1*002:01:01 would be simply “2”, while BF1*005:02:01 would be “5:02”), and then present the haplotype as a string in the order BLB1-BLB2-BF1-BF2. Thus, the B2 haplotype would be 2-2-2-2, the B6 haplotype 2-5-6-6, the B8 haplotype 2-8-5:02-5:02, and the B19 haplotype 12-12:02-12:02-15:02. An even greater simplification might be to name the BF-BL haplotypes after the BF2 genes (as has been done in Miller et al. 2004), but as “Bfbl haplotypes” to distinguish them from B haplotypes that describe the whole B locus. Thus, the haplotypes above would be named Bfbl 2, 6, 5:02 and 15:02.

## Application of the potential new nomenclature to other validated sequences from the literature

In order to extend the lists of alleles and haplotypes beyond the standard haplotypes, an extensive search of the general sequence database and of the scientific literature was carried out (Online Resources 1,2). Most of these sequences came from PCR amplifications of BLB exon 2 and of BF exon 2 to exon 3 from genomic DNA, subsequently cloned and sequenced. For some sequences, such amplifications from cDNA were also available. A few sequences were longer, but there are only two publications with sequences of complete genes, one from a line with a B6 haplotype (Suzuki et al. 2012) and the other from a line with a red junglefowl haplotype considered nearly identical with the B21 haplotype (Shiina et al. 2007). In general, evidence for multiple independent isolations (independent PCRs from a single chicken, more than one individual chicken or line, or from different laboratories) was required for the sequence to be considered valid.

**Fig. 5:**
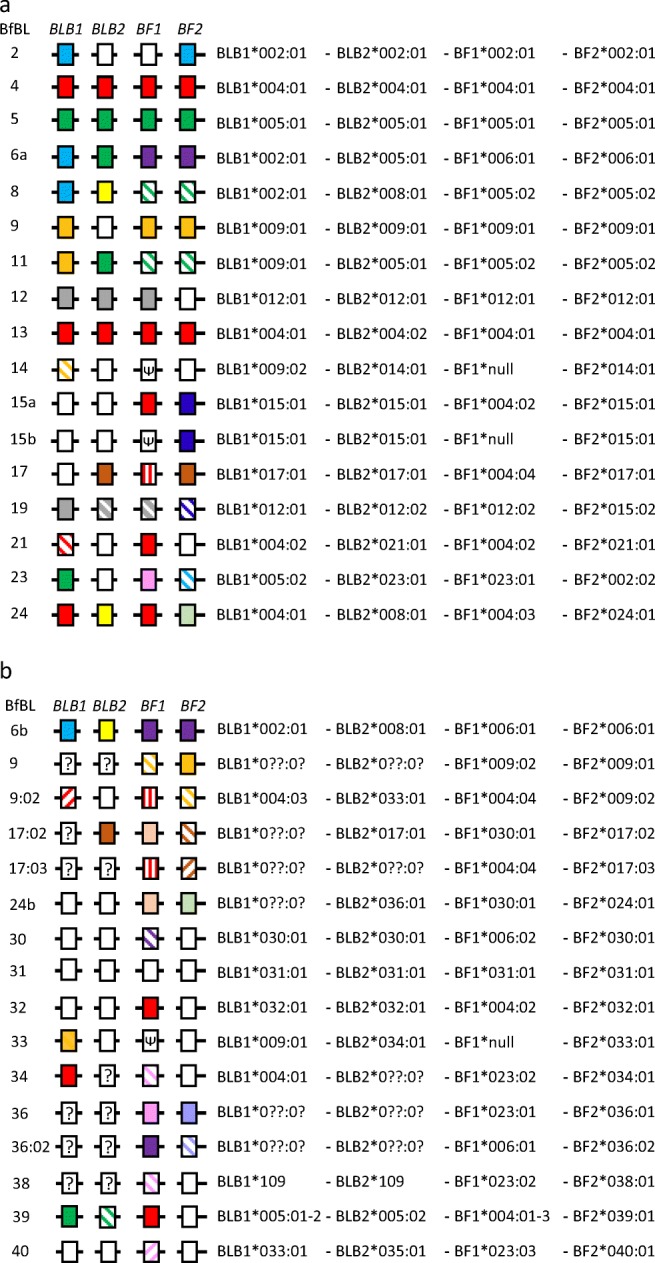
Haplotypes with genes as boxes, with haplotype strings of the potential allele names (with two fields). Comparisons are for peptide-binding domains/exons for **a** standard haplotypes, **b** sequences from the scientific literature. Unique sequences for each allelic series are indicated as white boxes, identical sequences in the coding region (CDS) are the same colours and closely related variants have the same colours but are striped in different ways for different variants of the same allele group. Note: in comparison to Fig. 4b, the boxes for some standard haplotypes are now coloured since the sequences, which were unique when only the standard haplotypes were compared, are now in an allelic group with sequences from the scientific literature. Also, some allele names are groups of closely related variants which cannot be distinguished by the peptide-binding domains/exons alone. GenBank accession numbers and citations for the sequences are in the legend of Online Resource 2

Only a few publications met the criteria of repeatability. Chief among them is a series of papers from the lab of Sandra Ewald (Li et al. 1997, 1999; Livant et al. 2001, 2004; supplemented with some direct submissions to GenBank for BLB1 sequences) that examined amplification of class I and class II B sequences from both cDNA and genomic DNA derived from lines of commercial broiler (that is, meat-type) chickens. A publication amplifying class I sequences from blue egg Caipira chickens in Brazil (Lima-Rosa et al. 2004) reported many of the same sequences. In addition, one publication reported class II B sequences from three Chinese lines (Chen et al. 2012) and another publication reported both class I and class II B sequences from a population of captive red junglefowl (Worley et al. 2008). Other publications and GenBank entries lacked replication, and for some sequences there was clear confusion between the publication and the associated database entries (for example, some sequences from Chen et al. 2012 and Worley et al. 2008). Once the sequences were considered valid, they were compared to those in the standard haplotypes (Fig. 5; Online Resource 2). Some of these sequences are identical or very similar to sequences in the standard haplotypes (leading to differences in the colours between Fig. 4b and Fig. 5a).

Some of these class I and class II B sequences were (or could be) assembled into BF-BL haplotypes, which will be referred to below by provisional names (Fig. 5; Online Resource 2). A few of these haplotypes are exactly as known for sequences from standard haplotypes, such as the Bfbl 2 (2-2-2-2) and 21 haplotypes (4-21-4:02-21). The gene sequences from the WLA line were reported as a B6 haplotype (Suzuki et al. 2012), but it is related to the standard haplotype from the line GB-2 haplotype (Hosomichi et al. 2008) by apparent recombination (2-8-6-6 compared to the standard haplotype 2-5-6-6), prompting a provisional name of Bflb 6b. It has not escaped the authors that such a name is not enormously different from the current method of naming recombinants with “r” and a number. Other full haplotypes assembled from sequences in the literature had BF2 alleles that are not found in the standard haplotypes (Fig. 5b). These include Bfbl 9:02 (4:03-33-4:04-9:02) and 30 (30-30-6:02-30) found in commercial and Brazilian chickens, 31 (31-31-31-31) found only in commercial chickens, 32 (32-32-4:02-32) found in commercial and wild chickens, 33 (9-34-null-33) in commercial, Brazilian and wild chickens, 38 [(109-109)-23:02-38], 39 (5-5:02-4-39) and 40 (33-35-23:03-40) found only in wild chickens.

For some studies in the literature, only partial haplotypes could be assembled or only single genes were reported. Partial haplotypes include Bfbl 9 (?-?- 9:02-9) from Brazilian chickens, 17:02 (?-17-30:01:02-17:02) from commercial and Brazilian chickens, 17:03 (?-?-4:04-17:03) from Brazilian chickens, 24b (?-36-30-24) from commercial chickens, 34 [4-?-23-34] in Brazilian and wild chickens, 36 (?-?-23-36) from commercial chickens, and 36:02 (?-?-6-36:02) from Brazilian chickens. Some singletons are identical to sequences from standard haplotypes, such as BF1*004:01:01, BF2*004:01:01, BF2*014:01:01 and BF2*015:01:01. Other singletons are closely related to standard sequences, such as BF2*015:03:01 from Brazilian chickens. Still others are not related to known sequences, such as BF2*035:01:01 found in commercial and Brazilian chickens and BF2*037:01:01 found in Brazilian chickens.

As the closely related sequences are reported to have been replicated, it is likely that they are real rather than some nucleotide mis-incorporation. All of the new genes and Bfbl haplotypes except for 36:02, 37, 39 and 40 have been extended and/or amply verified in a wide-ranging typing exercise of commercial egg-layers and broilers, fancy breeds and local (indigenous) chickens (C. Tregaskes, R. Martin, H. Afrache and J. Kaufman, unpublished).

Some of these new haplotypes highlighted unexpected difficulties, which have become ever more prominent in the wide-ranging typing exercise mentioned above. The Bfbl 33 haplotype lacks a BF1 allele (apparently, since absence of evidence is not evidence of absence), but this is no longer unexpected since standard B14 and B15 haplotypes also can lack a BF1 allele at both genomic and cDNA levels (Wallny et al. 2006; Shaw et al. 2007). However, the two BLB sequences could be found in clades with both BLB1 and BLB2 sequences; between gene PCRs established the locus for each of these alleles (Worley et al. 2008). More confusingly, the Bfbl 34 haplotype from red junglefowl has only one BLB sequence that is identical to BLB1*004:01:01, and between-gene PCRs located this sequence in the BLB1 locus (Worley et al. 2008). On the other hand, a Bfbl 34 haplotype has been found in the wide-ranging typing exercise that has two sequences neither of which is closely related to particular BLB sequences (C. Tregaskes, R. Martin, H. Afrache and J. Kaufman, unpublished), one located in the BLB1 locus and the other in the BLB2 locus (F. Filaire, H. Afrache, C. Tregaskes and J. Kaufman, unpublished). Similarly, the Bfbl 38 haplotype has only one BLB sequence which again is not closely related to any particular known BLB sequences, and between-gene PCRs show that this sequence is present in the BLB1 locus between Blec and tapasin as well as in the BLB2 locus between Brd2 and tapasin (Worley et al. 2008). Follow-up studies confirm that the same exon 2 sequence can be present in both the BLB1 and BLB2 loci (F. Filaire, R. Martin, H. Afrache, C. Tregaskes and J. Kaufman, unpublished), and a temporary assignment of such BLB sequences without a clear genetic location to numbers starting with 101 was established, along with the temporary use of curved parentheses to show that the location is unclear (such as “(109-109)” mentioned above). As discussed in the section about phylogenetic trees, BLB1 and BLB2 clades are more intermixed than BF1 and BF2 clades, which may reflect this phenomenon.

**Fig. 6:**
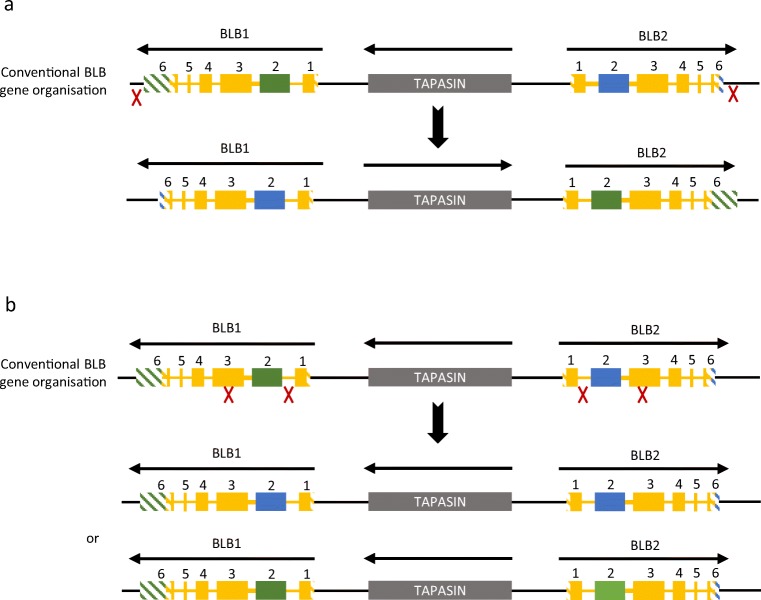
The compact nature of the BF-BL region and the inverted orientation of BLB1 and BLB2 can facilitate exchange between the two genes. Boxes indicate exons of BLB1 and BLB2, with solid colours indicating coding sequence and striped colours indicating untranslated regions. Yellow indicates sequence that is identical (or nearly so) between BLB1 and BLB2 genes; green and blue indicates regions that are specific to BLB1 and BLB2, respectively; a grey box indicates the tapasin gene. The conventional organisation is subject to sequence exchange by **a** simple inversion of the whole genes or **b** “gene conversion” (equivalent to double reciprocal recombination) of exon 2; red X indicate points of recombination and arrows above the genes indicate transcriptional orientation

It seems likely that the basis of the presence of (the same) BLB1 sequences in both the BLB1 and BLB2 genetic loci is due the compact nature of the BF-BL region and the fact that the BLB1 and BLB2 genes are in opposite transcriptional orientation. Gene conversion between homologous genes is thought to increase in frequency with decreasing physical distance between them (McCormack and Thompson 1990; Sayegh et al. 1999). Also, recombination between genes in opposite transcriptional orientation leads to inversion (Lundqvist et al. 2001; Zhao et al. 2000) rather than deletion as found for genes in the same orientation (Fig. 6).

## Discussion

An IPD-MHC database requires sequences that are validated and curated, but also requires a nomenclature that precisely allows the sequence to be identified in a convenient and biologically meaningful way. The system used for human MHC sequences (based on genetic loci, allele groups and variants within those groups) was adapted for the chicken MHC sequences long ago (Miller et al. 2004), but the allele names were based on traditional “standard” MHC haplotypes rather than on sequences. While recombination between haplotypes had been understood for many years (Simonsen et al. 1982), recombination among haplotypes is now known to have been too prevalent (Hosomichi et al. 2008) for a haplotype-based nomenclature to be sustained.

Some properties of the chicken MHC should make development of a nomenclature particularly easy. The BF-BL region is simple, with only two classical class I and two classical class II B genes that are polymorphic (Hosomichi et al. 2008; Jacob et al. 2000; Kaufman et al. 1999; Shaw et al. 2007; Wallny et al. 2006). Unlike some species, there is very little copy number variation (CNV): BF1 null alleles and a third BLB gene B12c found in some B12 haplotypes (Shaw et al. 2007; Zoorob et al. 1993). The β_2_-microglobulin and class II A (BLA) genes that encode the partner chains are non-polymorphic (Riegert et al. 1996; Salomonsen et al. 2003). The non-classical class I and class II B genes from the Rfp-Y region are sufficiently distant in sequence not to be amplified by typical PCR primers (Afanassieff et al. 2001; Zoorob et al. 1993). There is very little variation outside of the exons encoding the peptide-binding domains of the classical genes, including nearly invariant introns in between (Jacob et al. 2000; Shaw et al. 2007). Also, the compact nature of the BF-BL region, in which most introns are very small compared to most jawed vertebrates, is well-suited for PCR- and sequence-based typing methods (Potts 2016; C. Tregaskes, R. Martin, H. Afrache and J. Kaufman, unpublished).

However, the compact and simple nature of the chicken MHC also has disadvantages for analysis. In particular, the rarity of recombination leads to relatively stable haplotypes that encouraged a particularly simple nomenclature, but there is enough recombination (Hosomichi et al. 2008) to make this simple method untenable in the long run. On the flip side, the lack of recombination leads to difficulty in determining which gene within a haplotype is responsible for a biological trait. A newly appreciated difficulty is the exchange of information between the class II B genes (Worley et al. 2008), so that it may be difficult to assign a new sequence to a particular genetic locus. In fact, this kind of “concerted evolution” was first noticed long ago in the class II B genes of a closely related species, the pheasant (Wittzell et al. 1999). This exchange may be by gene conversion (which is increased by close proximity) or by inversion (which is possible due to the genes being in opposite transcriptional orientation) (McCormack and Thompson 1990; Lundqvist et al. 2001; Sayegh et al. 1999; Zhao et al. 2000). In comparison, such exchange of sequence between the class I genes seems rare, with only the BF1 genes of the B2 and B9 haplotypes looking very similar to BF2 alleles, including peptide-binding motif for BF1*002:01 (Chappell et al 2015). However, the presence of “B4 minor” class I sequences amplified preferentially from cDNA of the BA5 and BA12 haplotypes (Li et al. 1999) may mean that BF1*004 sequences can be highly expressed from the BF2 locus.

Having genes in opposite transcriptional orientation means that homologous recombination between them leads to inversion rather than loss of gene by deletion or CNV by unequal crossing-over (such as is seen for BG genes in the BG region, Salomonsen et al. 2014). Indeed, the BF-BL region has several such gene pairs in opposite transcriptional orientation (BLB1/BLB2, TAP1/TAP2, BF1/BF2, BNK/Blec), which may have evolved to avoid loss of genes from what has been characterised as a “minimal essential MHC” (Kaufman et al. 1995, 1999), which cannot afford to lose any “essential” genes.

The fact that the regulatory regions of these genes (for instance, promoters and 3’ untranslated regions) are generally separate from the coding regions and can be independently exchanged may have led to changes in expression that up to now have been considered to be a property of a particular locus (for instance, high and wide expression from the BLB1 and BF1 loci rather than from the BLB2 and BF2 loci). Although the compact nature of chicken MHC genes means that relatively simple sequencing can be performed for the exons encoding the peptide-binding regions (at least compared to many other animal species with long introns), it is not yet routine to sequence long stretches of DNA from many individual chickens, so locating the sequences to particular genetic loci and determining the regulation of those sequences remains a challenge. However, such determination would seem to be essential to ensure clear assignments in the database.

This exchange of information between genes also leads to difficulties in this new nomenclature system, which has as a central tenant that alleles are identified by sequence rather than by haplotype, so a unique sequence would have a unique name. For instance, the JF9 sequence was found in both the BLB1 and BLB2 loci of red junglefowl (Worley et al. 2008; F. Filaire, R. Martin, H. Afrache, C. Tregaskes and J. Kaufman, unpublished), so should these identical exon 2 sequences get different names? The typing exercise mentioned above (C. Tregaskes, R. Martin, H. Afrache and J. Kaufman, unpublished) has found many examples of similar sequences in both loci, so it is a real difficulty. One potential modification to ameliorate this confusion would be to give each sequence a unique name, perhaps ensuring that (as much as possible) those sequences predominantly found in BLB1 loci have odd numbers for alleles, and those found in BLB2 loci have even numbers for alleles. The extent to which this is feasible has yet to be ascertained. If such a modification is implemented for the class II B loci, then perhaps (in the interest of consistency) it should be considered for the class I loci, despite the lower level of exchange between the BF1 and BF2 genes.

Another unexpected concern involves the assignment of variants to alleles groups. For the standard haplotypes and the sequences from the literature, these assignments were almost unequivocal. However, as many new sequences have been encountered in the typing exercise mentioned above (C. Tregaskes, R. Martin, H. Afrache and J. Kaufman, unpublished), some clades have grown enormously, so that sequences within a clade can differ by more positions than sequences between clades. Apparently, this has also been a problem for the human MHC, for which the first allele groups were easily assigned based on serology, but wide-ranging sequencing led to enormous variation throughout the sequences, so that simple sequence comparisons began to be insufficient to make meaningful assignments (Robinson et al. 2017). Among the alternative possibilities would be a classification based on peptide-binding, if it can be related reliably to particular positions in the sequence, such as is attempted with the concept of supertypes (Greenbaum et al. 2011; Sidney et al. 2008).

In conclusion, the implementation of an IPD-MHC database for chicken MHC sequences has forced the consideration of a new nomenclature system based on gene sequences rather than on haplotypes, which is considered in this review. However, the discovery of many hundreds of new alleles in many new haplotypes has highlighted difficulties in this new naming process, the solutions to which are still under consideration. Therefore, the new names in this review should only be considered provisional at best, and may be replaced entirely in the future. Once a robust system has been worked out, then consultation with the avian immunology and genetics communities is envisaged. So overall, progress is being made, but problems have arisen. With the advent of easier methods for sequencing larger stretches of DNA, the next few years should see more complete sequences of the chicken MHC and hopefully the way forward will become clear.

## Electronic supplementary materials

ESM 1 (PDF 1882 kb)
