# Supplementary material for: A potential nomenclature for the Immuno Polymorphism Database (IPD) of chicken MHC genes: progress and problems
Source: Immunogenetics. 2019 Nov 18;72(1):9–24. doi: 10.1007/s00251-019-01145-6 (PMC6971145; doi:10.1007/s00251-019-01145-6)
Supplement: Supplementary file 1 — (PDF 1882 kb) [file 251_2019_1145_MOESM1_ESM.pdf]

Online Resources 1, 2, 3, 4, 5, and 6 for

A potential nomenclature for the immunopolymorphism database (IPD) of chicken MHC genes: progress and problems

Hassnae Afrache[1], Clive A. Tregaskes[1] and Jim Kaufman[1,2,*]

[1]University of Cambridge, Department of Pathology, Tennis Court Road, Cambridge, CB2 1QP, U. K.

[2]University of Cambridge, Department of Veterinary Medicine, Madingley Road, Cambridge, CB2 0ES

*corresponding author, jfk31@cam.ac.uk

current email addresses

Hassnae Afrache, ha395@cam.ac.uk
Clive A. Tregaskes, ct383@cam.ac.uk
Jim Kaufman, jfk31@cam.ac.uk

Key words: BF-BL region, BF1, BF2, BLB1, BLB2, recombination

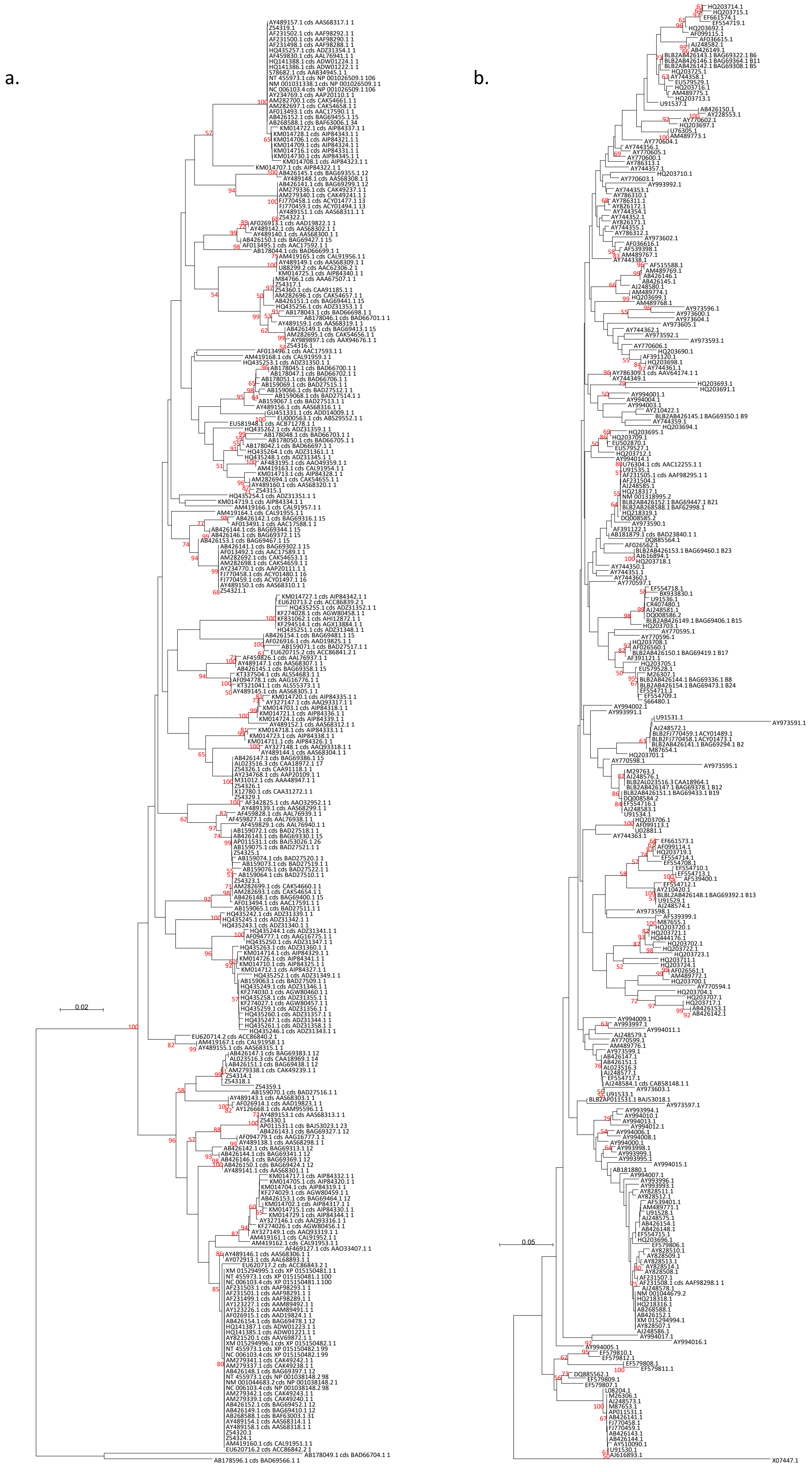
a.
b.
0.02
0.05

Online Resource 1. Phylogenetic trees for nucleotide sequences of exons encoding MHC peptide-binding domains from all chicken MHC-like sequences found in GenBank. a. exons 2 and 3 of chicken class I sequences; b. exon 2 of chicken class II B sequences. Sequences were found in two ways from the non-redundant GenBank database [National Center for Biotechnology Information (NCBI); www.ncbi.nlm.nih.gov/nuccore/] accessed in January 2017: by keywords (chicken AND MHC I AND gallus gallus, gallus gallus AND class I AND chromosome 16, g.gallus AND MHC class I, chicken AND MHC I AND gallus gallus AND chromosome 16), and by BLAST search using default parameters for nucleotide sequences (except using 1000 hits) and using AB426141 for BF and AB426141 for BLB as query sequences. Neighbour joining (NJ) trees were created by MEGA7 [Kumar S, Stecher G, Tamura K (2016) MEGA7: Molecular Evolutionary Genetics Analysis version 7.0 for bigger datasets. Molec Biol Evol 33:1870-1874] using Tamura-Nei method [Saitou N, Nei M (1987) The neighbor-joining method: A new method for reconstructing phylogenetic trees. Molec Biol Evol 4:406-425]. Genetic distances are indicated with bars; red numbers are bootstrap values (percentages) for those nodes that reach significance from 500 replications; names at the tips are the GenBank accession number (followed by the accession number for the CDS protein sequence of some entries)

New name	Old names	Accession numbers			References
BF1*002:01:01	**BF1*0201**	**AM279336**	**whole**	**gene**	**Shaw et al 2007**
	**BF1*0201**	**AM279340**	**whole**	**gene**	**Shaw et al 2007**
	**BF1*0201**	**AB426141**	**whole**	**gene**	**Hosomichi et al 2008**
	BFCC9b-BFCC9-2	AY489151-AY489174	partial	gene	Lima-Rosa et al 2004
	**B2m**	**Z54322**	**partial**	**cDNA**	**Wallny et al 2006**
BF1*004:01:01	**BF1*0401**	**AM279337**	**whole**	**gene**	**Shaw et al 2007**
	**BF1*0401**	**AM279341**	**whole**	**gene**	**Shaw et al 2007**
	**BF1*1301**	**AB426148**	**whole**	**gene**	**Hosomichi et al 2008**
	BF1*JF2	AM419160	partial	gene	Worley et al 2008
	**B4m**	**Z54324**	**partial**	**cDNA**	**Wallny et al 2006**
BF1*004:02:01	**BF1*1501**	**AB426149**	**whole**	**gene**	**Hosomichi et al 2008**
	**BF1*2101**	**AM279339**	**whole**	**gene**	**Shaw et al 2007**
	**BF1*2101**	**AM279342**	**whole**	**gene**	**Shaw et al 2007**
	**BF1*2101**	**AB426152**	**whole**	**gene**	**Hosomichi et al 2008**
	**BF1*21**	**AY821520**	**whole**	**cDNA**	**Dalgaard et al 2005**
	BF1*W1	HQ141385	whole	cDNA	Kjaerup and Juul-Madsen, direct submission 2010
	BF1*131	HQ141387	whole	cDNA	Kjaerup and Juul-Madsen, direct submission 2010
	BF*C5b-BF*C5b	AY123227-AY128692	partial	gene	Livant et al 2004
	BFCC2b-BFCC15-2	AY489158-AY489181	partial	gene	Lima-Rosa et al 2004
	BFCC14b-BFCC12	AY489154-AY489177	partial	gene	Lima-Rosa et al 2004
	B-F200minor	AF231503	partial	cDNA	Juul-Madsen et al 2000
	B-F201minor	AF231501	partial	cDNA	Juul-Madsen et al 2000
	B-FW1minor	AF231499	partial	cDNA	Juul-Madsen et al 2000
	BA12	AF026915	partial	cDNA	Li et al 1999
	**B21m**	**Z54320**	**partial**	**cDNA**	**Wallny et al 2006**
BF1*004:03:01	**BF1*2401**	**AB426154**	**whole**	**gene**	**Hosomichi et al 2008**
BF1*004:04:01	**BF1*1701**	**AB426150**	**whole**	**gene**	**Hosomichi et al 2008**
	BFCC3b-BFCC3-2	AY489141-AY489164	partial	gene	Lima-Rosa et al 2004
BF1*004:05:01	BFCC6b-BFCC6-2	AY489146-AY489169	partial	gene	Lima-Rosa et al 2004
	BF*C7b	AY072913	partial	gene	Livant et al 2004
BF1*005:01:01	**BF1*0501**	**AB426142**	**whole**	**gene**	**Hosomichi et al 2008**
BF1*005:02:01	**BF1*0801**	**AB426144**	**whole**	**gene**	**Hosomichi et al 2008**
	**BF1*1101**	**AB426146**	**whole**	**gene**	**Hosomichi et al 2008**
BF1*006:01:01	**BF1*0601**	**AB426143**	**whole**	**gene**	**Hosomichi et al 2008**
	**BF6M**	**Z54330**	**partial**	**cDNA**	**Wallny et al 2006**
	BFCC10b-BFCC11	AY489153-AY489176	partial	gene	Lima-Rosa et al 2004
BF1*006:02:01	BF*C1b	AF094779	partial	gene	Livant et al 2004
	BFCC1b-BFCC1	AY489138-AY489161	partial	gene	Lima-Rosa et al 2004
BF1*009:01:01	**BF1*0901**	**AB426145**	**whole**	**gene**	**Hosomichi et al 2008**
BF1*009:02:01	BF*CC7b-BFCC7-2	AY489148-AY489171	partial	gene	Lima-Rosa et al 2004
BF1*012:01:01	**BF2*1202**	**AL023516**	**whole**	**gene**	**Kaufman et al 1999**
	**BF1*1201**	**AB426147**	**whole**	**gene**	**Shiina et al 2007**
	**BF12m**	**Z54314 with T4S**	**partial**	**cDNA**	**Wallny et al 2006**
BF1*012:02:01	**BF1*1902**	**AM279338**	**whole**	**gene**	**Shaw et al 2007**
	**BF1*1901**	**AB426151**	**whole**	**gene**	**Hosomichi et al 2008**
	**BF19m**	**Z54318 with T4S**	**partial**	**cDNA**	**Wallny et al 2006**
BF1*023:01:01	**BF1*2301**	**AB426153**	**whole**	**gene**	**Hosomichi et al 2008**
	BF*H8b	AY327146	partial	gene	Livant et al 2004
BF1*023:02:01	BF1*JF4	AM419161	partial	gene	Worley et al 2008
BF1*023:03:01	BF1*JF6	AM419162	partial	gene	Worley et al 2008
BF1*030:01:01	BA9-1	AF026914	partial	gene	Li et al 1999
	BF*A1b	AY126668	partial	gene	Livant et al 2004
	BFCC4b-BFCC4-2	AY489143-AY489166	partial	gene	Lima-Rosa et al 2004
BF1*031:01:01	BF*C2vb	AF469127	partial	gene	Livant et al 2004

Online Resource 2. Tables of potential BF and BLB names with accession numbers and citations for standard haplotypes and literature alleles; bold entries are for sequences from the standard haplotypes (page 1 of 8).

New name	Old names	Accession numbers			References
BF2*002:01:01	**BF2*0201**	**AM282692**	**whole**	**gene**	**Shaw et al 2007**
	**BF2*0201**	**AM282698**	**whole**	**gene**	**Shaw et al 2007**
	**BF2*0201**	**AB426141**	**whole**	**gene**	**Hosomichi et al 2008**
	BFIV2	AF013492	whole	cDNA	Hunt and Fulton 1998
	BFCC9a-BFCC9-1	AY489150-AY489173	partial	gene	Lima-Rosa et al 2004
	**B2M**	**Z54321**	**partial**	**cDNA**	**Wallny et al 2006**
BF2*002:02:01	**BF2*2301**	**AB426153**	**whole**	**gene**	**Hosomichi et al 2008**
BF2*004:01:01	**BF2*0401**	**AM282693**	**whole**	**gene**	**Shaw et al 2007**
	**BF2*0401**	**AM282699**	**whole**	**gene**	**Shaw et al 2007**
	**BF2*1301**	**AB426148**	**whole**	**gene**	**Hosomichi et al 2008**
	**B4M**	**Z54323**	**partial**	**cDNA**	**Wallny et al 2006**
BF2*005:01:01	**BF2*0501**	**AB426142**	**whole**	**gene**	**Hosomichi et al 2008**
	**BFIV5**	**AF013491**	**whole**	**cDNA**	**Hunt and Fulton 1998**
BF2*005:02:01	**BF2*0801**	**AB426144**	**whole**	**gene**	**Hosomichi et al 2008**
	**BF2*1101**	**AB426146**	**whole**	**gene**	**Hosomichi et al 2008**
BF2*006:01:01	**BF2*0601**	**AB426143**	**whole**	**gene**	**Hosomichi et al 2008**
	**BF6m**	**Z54325**	**partial**	**cDNA**	**Wallny et al 2006**
BF2*009:01:01	**BF2*0901**	**AB426145**	**whole**	**gene**	**Hosomichi et al 2008**
	BFCC7a-BFCC7-1	AY489147-AY489170	partial	gene	Lima-Rosa et al 2004
	Fayoumi	AF459826	partial	gene	Liu et al 2002
BF2*009:02:01	BFCC6a-BFCC6-1	AY489145-AY489168	partial	gene	Lima-Rosa et al 2004
	BC7	AF094778	partial	cDNA	Livant et al 2001
BF2*012:01:01	**B12**	**M31012**	**whole**	**gene**	**Kroemer et al 1990**
	**BF1*1201**	**AL023516**	**whole**	**gene**	**Kaufman et al 1999**
	**BF2*1201**	**AB426147**	**whole**	**gene**	**Hosomichi et al 2008**
	**F10**	**X12780**	**whole**	**cDNA**	**Guillemot 1988**
	**BF12M**	**Z54329**	**partial**	**cDNA**	**Wallny et al 2006**
BF2*014:01:01	**BF2*1401**	**AM282694**	**whole**	**gene**	**Shaw et al 2007**
	BFCC12a-BFCC17	AY489160-AY489183	partial	gene	Lima-Rosa et al 2004
BF2*015:01:01	**BF2*1501**	**AM282695**	**whole**	**gene**	**Shaw et al 2007**
	**BF2*1501**	**AB426149**	**whole**	**gene**	**Hosomichi et al 2008**
	**BFIV15**	**L28958**	**whole**	**cDNA**	**Hunt et al 1994**
	**BF15M**	**Z54316**	**partial**	**cDNA**	**Wallny et al 2006**
BF2*015:02:01	**BF2*1902**	**AM282696**	**whole**	**gene**	**Shaw et al 2007**
	**BF2*1901**	**AB426151**	**whole**	**gene**	**Hosomichi et al 2008**
	**B19**	**M84766**	**whole**	**cDNA**	**Kaufman et al 1992**
	**BF19M**	**Z54317**	**partial**	**cDNA**	**Wallny et al 2006**
BF2*015:03:01	BFCC11a-BFCC16	AY489159-AY489182	partial	gene	Lima-Rosa et al 2004
BF2*017:01:01	**BF2*1701**	**AB426150**	**whole**	**gene**	**Hosomichi et al 2008**
	**BFIV17**	**AF013495**	**whole**	**cDNA**	**Hunt and Fulton 1998**
BF2*017:02:01	BA1-1	AF026913	partial	cDNA	Li et al 1999
	BFCC4a-BFCC4-1	AY489142-AY489165	partial	gene	Lima-Rosa et al 2004
BF2*017:03:01	BFCC3a-BFCC3-1	AY489140-AY489163	partial	gene	Lima-Rosa et al 2004
BF2*018:01:01	**BFIV18**	**AF013496**	**whole**	**cDNA**	**Hunt and Fulton 1998**
BF2*021:01:01	**BF2*2101**	**AM282697**	**whole**	**gene**	**Shaw et al 2007**
	**BF2*2101**	**AM282700**	**whole**	**gene**	**Shaw et al 2007**
	**BF2*2101**	**AB426152**	**whole**	**gene**	**Hosomichi et al 2008**
	**BFIV21**	**S78682**	**whole**	**cDNA**	**Fulton et al 1995**
	**BFIV21**	**AF013493**	**whole**	**cDNA**	**Hunt and Fulton 1998**
	BF2*131	HQ141388	whole	cDNA	Kjaerup and Juul-Madsen, direct submission 2010
	BF2*W1	HQ141386	whole	cDNA	Kjaerup and Juul-Madsen, direct submission 2010
	BFCC2a-BFCC15-1	AY489157-AY489180	partial	gene	Lima-Rosa et al 2004
	Spanish	AF459830	partial	cDNA	Liu et al 2002
	**BF21M**	**Z54319**	**partial**	**cDNA**	**Wallny et al 2006**
BF2*024:01:01	**BF2*2401**	**AB426154**	**whole**	**gene**	**Hosomichi et al 2008**
	BA9-2	AF026916	partial	cDNA	Li et al 1999

New name	Old names	Accession numbers			References
BF2*030:01:01	BF*C1a	AF342825	partial	gene	Livant et al 2004
	BFCC1a-BFCC2a	AY489139-AY489162	partial	gene	Lima-Rosa et al 2004
BF2*031:01:01	BC2v	AF094777	partial	gene	Livant et al 2001
BF2*032:01	BF*A12a	AF483195	partial	gene	Livant et al 2004
	BF2*JF1	AM419163	partial	gene	Worley et al 2008
BF2*033:01:01	BFCC8a-BFCC8	AY489149-AY489172	partial	gene	Lima-Rosa et al 2004
	BF2*JF5	AM419165	partial	gene	Worley et al 2008
	BA4var	U88299	partial	cDNA	Li et al 1999
BF2*034:01:01	BFCC13a-BFCC13	AY489155-AY489178	partial	gene	Lima-Rosa et al 2004
	BF2*JF8	AM419167	partial	gene	Worley et al 2008
BF2*035:01:01	BF*J3a	AY327148	partial	gene	Livant et al 2004
	BFCC5a-BFCC5	AY489144-AY489167	partial	gene	Lima-Rosa et al 2004
BF2*036:01:01	BF*H8a	AY327147	partial	gene	Livant et al 2004
BF2*036:02:01	BFCC10a-BFCC10	AY489152-AY489175	partial	gene	Lima-Rosa et al 2004
BF2*037:01:01	BFCC14a-BFCC14	AY489156-AY489179	partial	gene	Lima-Rosa et al 2004
BF2*038:01:01	BF2*JF3	AM419164	partial	gene	Worley et al 2008
BF2*039:01:01	BF2*JF7	AM419166	partial	gene	Worley et al 2008
BF2*040:01:01	BF2*JF9	AM419168	partial	gene	Worley et al 2008

Online Resource 2, continued (page 3 of 8).

New name	Old name	Accession number			References
BLB1*002:01:01	**BLB1-B2**	**AB426141.1**	**whole**	**gene**	**Hosomichi et al 2008**
	**BLB1-BR4**	**FJ770458.1**	**whole**	**gene**	**Goto et al 2009**
	**BLB1-BR2**	**FJ770459.1**	**whole**	**gene**	**Goto et al 2009**
	**B-LB2 minor**	**AJ248573.1**	**partial**	**gene**	**Jacob et al 2000**
	**B2b_B-LBI**	**U91530.1**	**partial**	**cDNA**	**Pharr et al 1998**
	**BLB1-B8**	**AB426144.1**	**whole**	**gene**	**Hosomichi et al 2008**
	BLB1 WLA	AP011531.1	whole	gene	Suzuki et al 2012
BLB1*002:01:02	**BLB1-B6**	**AB426143.1**	**whole**	**gene**	**Hosomichi et al 2008**
BLB1*004:01:01	**B-LB4 minor**	**AJ248575.1**	**partial**	**gene**	**Jacob et al 2000**
	**BLB1-B13**	**AB426148.1**	**whole**	**gene**	**Hosomichi et al 2008**
	**BLB1-B24**	**AB426154.1**	**whole**	**gene**	**Hosomichi et al 2008**
	BLB1*JF10	AM489771.1	partial	gene	Worley et al 2008
BLB1*004:02:01	**BLB1-B21**	**AB426152.1**	**whole**	**gene**	**Hosomichi et al 2008**
	**B-LB21 minor**	**AJ248586.1**	**partial**	**gene**	**Jacob et al 2000**
	**BLBI**	**AB268588.1**	**whole**	**gene**	**Shiina et al 2007**
	BLB1*131	HQ218316.1	partial	cDNA	Kjaerup and Juul-Madsen, direct submission 2010
	BLB1*W1	HQ218318.1	partial	cDNA	Kjaerup and Juul-Madsen, direct submission 2010
	GSP-BLB1	AB181880.1	partial	cDNA	Hosomichi, direct submission 2004
BLB1*004:03:01	B-L beta minor-BC7	AF539401.1	partial	gene	Livant and Ewald, direct submission 2002
BLB1*004:04:01	B-LW1minor	AF231507.1	partial	cDNA	Juul-Madsen et al 2000
	B200minor	AF231508.1	partial	cDNA	Juul-Madsen et al 2000
BLB1*005:01:01	**BLB1-B5**	**AB426142.1**	**whole**	**gene**	**Hosomichi et al 2008**
	BLB1*JF9	AM489770.1	partial	gene	Worley et al 2008
BLB1*005:02:01	**BLB1-B23**	**AB426153.1**	**whole**	**gene**	**Hosomichi et al 2008**
BLB1*005:03:01	B-L beta minor-BA9	AY702658.1	partial	gene	Livant and Ewald, direct submission 2004
BLB1*009:01:01	**BLB1-B9**	**AB426145.1**	**whole**	**gene**	**Hosomichi et al 2008**
	BLB1*JF7	AM489769.1	partial	gene	Worley et al 2008
	B-L beta minor-BA4v	AF515588.1	partial	gene	Livant and Ewald, direct submission 2002
BLB1*009:01:02	**BLB1-B11**	**AB426146.1**	**whole**	**gene**	**Hosomichi et al 2008**
BLB1*009:02:01	**B-LB14 minor**	**AJ248580.1**	**partial**	**gene**	**Jacob et al 2000**
BLB1*012:01:01	**BLb1**	**AL023516.3**	**whole**	**gene**	**Kaufman et al 1999**
	**BLB1-B12**	**AB426147.1**	**whole**	**gene**	**Hosomichi et al 2008**
	**B-LB12 minor**	**AJ248577.1**	**partial**	**gene**	**Jacob et al 2000**
	**BLB1-B19**	**AB426151.1**	**whole**	**gene**	**Hosomichi et al 2008**
	**B-LB19 minor**	**AJ248584.1**	**partial**	**gene**	**Jacob et al 2000**
	**B19a_B-LBI**	**U91533.1**	**partial**	**cDNA**	**Pharr et al 1998**
BLB1*015:01:01	**BLB1-B15**	**AB426149.1**	**whole**	**gene**	**Hosomichi et al 2008**
	**B-LB15 minor**	**AJ248582.1**	**partial**	**gene**	**Jacob et al 2000**
BLB1*015:02:01	BA11-b allele	AF036615.1	partial	gene	Zheng et al 1999
BLB1*017:01:01	**BLB1-B17**	**AB426150.1**	**whole**	**gene**	**Hosomichi et al 2008**
	B-L beta minor-BA1	AY228553.1	partial	gene	Livant and Ewald, direct submission 2003
BLB1*030:01:01	B-L beta	M87655.1	whole	cDNA	Pharr et al 1993
	B-L beta minor-BC1	AF539399.1	partial	gene	Livant and Ewald, direct submission 2002
BLB1*031:01:01	B-L beta minor-BC2v	AF539400.1	partial	gene	Livant and Ewald, direct submission 2002
BLB1*032:01:01	BLB1*JF2	AM489767.1	partial	gene	Worley et al 2008
	B-L beta minor-BA12	AF539398.1	partial	gene	Livant and Ewald, direct submission 2002
BLB1*032:02:01	BA12-b allele	AF036616.1	partial	gene	Zheng et al 1999
BLB1*033:01:01	BLB1*JF5	AM489768.1	partial	gene	Worley et al 2008
BLB*109	BLB*JF3	AM489776.1	partial	gene	Worley et al 2008

Online Resource 2, continued (page 4 of 8).

New name	Old name	Accession number			References
BLB2*002:01:01	**BLB-B2**	**AB426141.1**	**whole**	**gene**	**Hosomichi et al 2008**
	**BLB2-BR4**	**FJ770458.1**	**whole**	**gene**	**Goto et al 2009**
	**BLB2-BR2**	**FJ770459.1**	**whole**	**gene**	**Goto et al 2009**
	**B-LB2 major**	**AJ248572.1**	**partial**	**gene**	**Jacob et al 2000**
	**B2a_B-LBII**	**U91531.1**	**partial**	**cDNA**	**Pharr et al 1998**
BLB2*004:01:01	**B-LB4 major**	**AJ248574.1**	**partial**	**gene**	**Jacob et al 2000**
BLB2*004:02:01	**BLB2-B13**	**AB426148.1**	**whole**	**gene**	**Hosomichi et al 2008**
	**B13a_B-LBII**	**U91529.1**	**partial**	**cDNA**	**Pharr et al 1998**
	B50 GB-1	AY210420.1	partial	gene	Zhou and Lamont 2003
BLB2*005:01:01	**BLB2-B5**	**AB426142.1**	**whole**	**gene**	**Hosomichi et al 2008**
	**BLB2-B6**	**AB426143.1**	**whole**	**gene**	**Hosomichi et al 2008**
	**B11**	**AB426146.1**	**whole**	**gene**	**Hosomichi et al 2008**
BLB2*005:02:01	BLB2*JF8	AM489775.1	partial	gene	Worley et al 2008
BLB2*005:03:01	B-LB2*1001	AY744358.1	partial	gene	Xu et al 2007
BLB2*008:01:01	**BLB2-B8**	**AB426144.1**	**whole**	**gene**	**Hosomichi et al 2008**
	BLB2 WLA	AP011531.1	whole	gene	Suzuki et al 2012
BLB2*008:01:02	**BLB2-B24**	**AB426154.1**	**whole**	**gene**	**Hosomichi et al 2008**
BLB2*009:01:01	**BLB2-B9**	**AB426145.1**	**whole**	**gene**	**Hosomichi et al 2008**
BLB2*012:01:01	**BLb2**	**AL023516.3**	**whole**	**gene**	**Kaufman et al 1999**
	**BLB2-B12**	**AB426147.1**	**whole**	**gene**	**Hosomichi et al 2008**
	**B-LBII-beta**	**M29763.1**	**whole**	**gene**	**Zoorob et al 1990**
	**B-LB12 major**	**AJ248576.1**	**partial**	**gene**	**Jacob et al 2000**
BLB2*012:02:01	**BLB-B19**	**AB426151.1**	**whole**	**gene**	**Hosomichi et al 2008**
	**B-LB-B19**	**DQ008584.2**	**whole**	**cDNA**	**Niemiec et al 2006**
	**B-LB19 major**	**AJ248583.1**	**partial**	**gene**	**Jacob et al 2000**
	**B19b_B-LBII**	**U91534.1**	**partial**	**cDNA**	**Pharr et al 1998**
BLB2*014:01:01	**B-LB14 major**	**AJ248579.1**	**partial**	**gene**	**Jacob et al 2000**
BLB2*015:01:01	**B15**	**AB426149.1**	**whole**	**gene**	**Hosomichi et al 2008**
	**B-LB15 major**	**AJ248581.1**	**partial**	**gene**	**Jacob et al 2000**
	**B-LB-15I5**	**DQ008586.2**	**whole**	**cDNA**	**Niemiec and Sharif, direct submission 2005**
BLB2*017:01:01	**BLB2-B17**	**AB426150.1**	**whole**	**gene**	**Hosomichi et al 2008**
	B-L beta allele A1	AF026560.1	partial	cDNA	Li et al 1999
BLB2*021:01:01	**B21**	**AB426152.1**	**whole**	**gene**	**Hosomichi et al 2008**
	**BLBII**	**AB268588.1**	**whole**	**gene**	**Shiina et al 2007**
	**B-LB21 major**	**AJ248585.1**	**partial**	**gene**	**Jacob et al 2000**
	**B21_B-LBII**	**U91535.1**	**partial**	**cDNA**	**Pharr et al 1998**
	BLB2*131	HQ218317.1	partial	cDNA	Kjaerup and Juul-Madsen, direct submission 2010
	BLB2*W1	HQ218319.1	partial	cDNA	Kjaerup and Juul-Madsen, direct submission 2010
	B-LW1major	AF231504.1	partial	cDNA	Juul-Madsen et al 2000
	B-L201major	AF231505.1	partial	cDNA	Juul-Madsen et al 2000
	B-L200major	AF231506.1	partial	cDNA	Juul-Madsen et al 2000
	BA4	U76304.1	partial	cDNA	Li et al 1997
	GSP-BLB2	AB181879.1	partial	cDNA	Hosomichi, direct submission 2004
BLB2*023:01:01	**BLB2-B23**	**AB426153.1**	**whole**	**gene**	**Hosomichi et al 2008**
BLB2*030:01:01	BC1	AF099113.1	partial	cDNA	Livant et al 2001
	B-L beta SPAFAS line 11	U02881.1	partial	cDNA	Pharr, direct submission 1993
	B-LB BQ355	HQ203706.1	whole	cDNA	Chen et al 2012
BLB2*031:01:01	BC2v	AF099114.1	partial	cDNA	Livant et al 2001
	B-LB BH478	HQ203719.1	whole	cDNA	Chen et al 2012
BLB2*032:01:01	BA12	AF026561.1	partial	cDNA	Li et al 1999
	BLB2*JF1	AM489772.1	partial	gene	Worley et al 2008
BLB2*033:01:01	BL-beta BC7	AF099115.1	partial	cDNA	Livant et al 2001
BLB2*034:01:01	BA4v	U76305.1	partial	cDNA	Li et al 1997
	BLB2*JF4	AM489773.1	partial	gene	Worley et al 2008
BLB2*035:01:01	BLB2*JF6	AM489774.1	partial	gene	Worley et al 2008
	B-LB BW462	HQ203699.1	whole	cDNA	Chen et al 2012
BLB2*036:01:01	BA9	AF026562.1	whole	cDNA	Li et al 1999
BLB*109	BLB*JF3	AM489776.1	partial	gene	Worley et al 2008

Online Resource 2, continued (page 5 of 8).

Citations:

Chen F, Pan L, Chao W, Dai Y, Yu W (2012) Character of chicken polymorphic major histocompatibility complex class II alleles of 3 Chinese local breeds. Poult Sci 91:1097-1104. doi: 10.3382/ps.2011-02007. PubMed PMID: 22499866.

Dalgaard TS, Vitved L, SkjÃ¸dt K, Thomsen B, Labouriau R, Jensen KH,

Juul-Madsen HR (2005) Molecular characterization of major histocompatibility complex class I (B-F) mRNA variants from chickens differing in resistance to Marek's disease. Scand J Immunol 62:259-270. PubMed PMID: 16179013

Fulton JE, Thacker EL, Bacon LD, Hunt HD (1995) Functional analysis of avian class I (BFIV) glycoproteins by epitope tagging and mutagenesis in vitro. Eur J Immunol 25:2069-2076. PubMed PMID: 7621880.

Goto RM, Wang Y, Taylor RL Jr, Wakenell PS, Hosomichi K, Shiina T, Blackmore

CS, Briles WE, Miller MM (2009) BG1 has a major role in MHC-linked resistance to malignant lymphoma in the chicken. Proc Natl Acad Sci U S A 106:16740-16745. doi: 10.1073/pnas.0906776106.PubMed PMID: 19805366; PubMedCentral PMCID: PMC2757851.

Guillemot F, Billault A, Pourquié O, Béhar G, Chaussé AM, Zoorob R, Kreibich

G, Auffray C. (1998) A molecular map of the chicken major histocompatibility complex: the class II beta genes are closely linked to the class I genes and the nucleolar organizer. EMBO J 7:2775-2785. PubMed PMID: 3141149; PubMed CentralPMCID: PMC457068.

Hosomichi K, Miller MM, Goto RM, Wang Y, Suzuki S, Kulski JK, Nishibori M,Inoko H, Hanzawa K, Shiina T (2008) Contribution of mutation, recombination, and gene conversion to chicken MHC-B haplotype diversity. J Immunol 181:3393-3399. PubMed PMID: 18714011; PubMed Central PMCID: PMC2657362.

Hunt HD, Fulton JE (1998) Analysis of polymorphisms in the major expressed class I locus (B-FIV) of the chicken. Immunogenetics 47:456-467. PubMed PMID: 9553152.

Hunt HD, Pharr GT, Bacon LD (1994) Molecular analysis reveals MHC class I intra-locus recombination in the chicken. Immunogenetics 40:370-375. PubMed PMID: 7927541.

Jacob JP, Milne S, Beck S, Kaufman J (2000) The major and a minor class II beta-chain (B-LB) gene flank the Tapasin gene in the B-F /B-L region of the chicken major histocompatibility complex. Immunogenetics 51:138-147. PubMed PMID: 10663576.

Juul-Madsen HR, Dalgaard TS, Afanassieff M (2000) Molecular characterization of major and minor MHC class I and II genes in B21-like haplotypes in chickens. Anim Genet 31:252-261. PubMed PMID: 11086534.

Kaufman J, Andersen R, Avila D, Engberg J, Lambris J, Salomonsen J, Welinder K, Skjodt K (1992) Different features of the MHC class I heterodimer have evolved at different rates. Chicken B-F and beta 2-microglobulin sequences reveal invariant surface residues. J Immunol 148:1532-1546. PubMed PMID: 1538136.

Kaufman J, Milne S, Gobel TW, Walker BA, Jacob JP, Auffray C, Zoorob R, Beck S (1999) The chicken B locus is a minimal essential major histocompatibility complex. Nature 401:923-925. PubMed PMID: 10553909.

Kroemer G, Zoorob R, Auffray C (1990) Structure and expression of a chicken MHC class I gene. Immunogenetics 31:405-409. PubMed PMID: 2370087.

Li L, Johnson LW, Ewald SJ (1997) Molecular characterization of major histocompatibility complex (B) haplotypes in broiler chickens. Anim Genet 28:258-267. PubMed PMID: 9345722.

Li L, Johnson LW, Livant EJ, Ewald SJ (1999) The MHC of a broiler chicken line: serology, B-G genotypes, and B-F/B-LB sequences. Immunogenetics 49:215-224. PubMed PMID: 9914335.

Lima-Rosa CA, Canal CW, Streck AF, Freitas LB, Delgado-Canedo A, Bonatto SL, Salzano FM (2004) B-F DNA sequence variability in Brazilian (blue-egg Caipira) chickens. Anim Genet 35:278-284. PubMed PMID: 15265066.

Liu W, Miller MM, Lamont SJ (2002) Association of MHC class I and class II gene polymorphisms with vaccine or challenge response to Salmonella enteritidis in young chicks. Immunogenetics 54:582-590. PubMed PMID: 12439621.

Livant EJ, Zheng D, Johnson LW, Shi W, Ewald SJ (2001) Three new MHC haplotypes in broiler breeder chickens. Anim Genet 32:123-131. PubMed PMID: 11493260.

Livant EJ, Brigati JR, Ewald SJ (2004) Diversity and locus specificity of chicken MHC B class I sequences. Anim Genet 35:18-27. PubMed PMID: 14731225.

Niemiec PK, Read LR, Sharif S. (2006) Synthesis of chicken major histocompatibility complex class II oligomers using a baculovirus expression system. Protein Expr Purif 46:390-400. PubMed PMID: 16236525.

Pharr GT, Bacon LD, Dodgson JB (1993) Analysis of B-L beta-chain gene expression in two chicken cDNA libraries. Immunogenetics 37:381-385. PubMed PMID:8428771.

Pharr GT, Dodgson JB, Hunt HD, Bacon LD (1998) Class II MHC cDNAs in 15I5 B-congenic chickens. Immunogenetics 47:350-354. PubMed PMID: 9510552.

Shaw I, Powell TJ, Marston DA, Baker K, van Hateren A, Riegert P, Wiles MV, Milne S, Beck S, Kaufman J (2007) Different evolutionary histories of the two classical class I genes BF1 and BF2 illustrate drift and selection within the stable MHC haplotypes of chickens. J Immunol 178:5744-5752. PubMed PMID: 17442958.

Shiina T, Briles WE, Goto RM, Hosomichi K, Yanagiya K, Shimizu S, Inoko H, Miller MM (2007) Extended gene map reveals tripartite motif, C-type lectin, and Ig superfamily type genes within a subregion of the chicken MHC-B affecting infectious disease. J Immunol 178:7162-7172. PubMed PMID: 17513765.

Suzuki K, Kobayashi E, Yamashita H, Uenishi H, Churkina I, Plastow G, Hamasima N, Mitsuhashi T (2012) Structural analysis of MHC alleles in an RSV tumour regression chicken using a BAC library. Anim Genet 43:348-351. doi: 10.1111/j.1365-2052.2011.02247.x. PubMed PMID: 22486511.

Wallny HJ, Avila D, Hunt LG, Powell TJ, Riegert P, Salomonsen J, Skjodt K, Vainio O, Vilbois F, Wiles MV, Kaufman J (2006) Peptide motifs of the single dominantly expressed class I molecule explain the striking MHC-determined response to Rous sarcoma virus in chickens. Proc Natl Acad Sci U S A 103:1434-1439. PubMed PMID: 16432226; PubMed Central PMCID: PMC1360531.

Worley K, Gillingham M, Jensen P, Kennedy LJ, Pizzari T, Kaufman J, Richardson DS (2008) Single locus typing of MHC class I and class II B loci in a population of red jungle fowl. Immunogenetics 60:233-247. doi: 10.1007/s00251-008-0288-0. PubMed PMID: 18389232.

Xu R, Li K, Chen G, Xu H, Qiang B, Li C, Liu B (2007) Characterization of genetic polymorphism of novel MHC B-LB II alleles in Chinese indigenous chickens. J Genet Genomics 34:109-118. PubMed PMID: 17469783.

Zheng D, O'Keefe G, Li L, Johnson LW, Ewald SJ (1999) A PCR method for typing B-L beta II family (class II MHC) alleles in broiler chickens. Anim Genet 30:109-119. PubMed PMID: 10376301.

Zhou H, Lamont SJ (2003) Chicken MHC class I and II gene effects on antibody response kinetics in adult chickens. Immunogenetics 55:133-140. doi: 10.1007/s00251-003-0566-9. PubMed PMID: 12743657.

Zoorob R, Béhar G, Kroemer G, Auffray C (1990) Organization of a functional chicken class II B gene. Immunogenetics 31:179-187. PubMed PMID: 1969383.

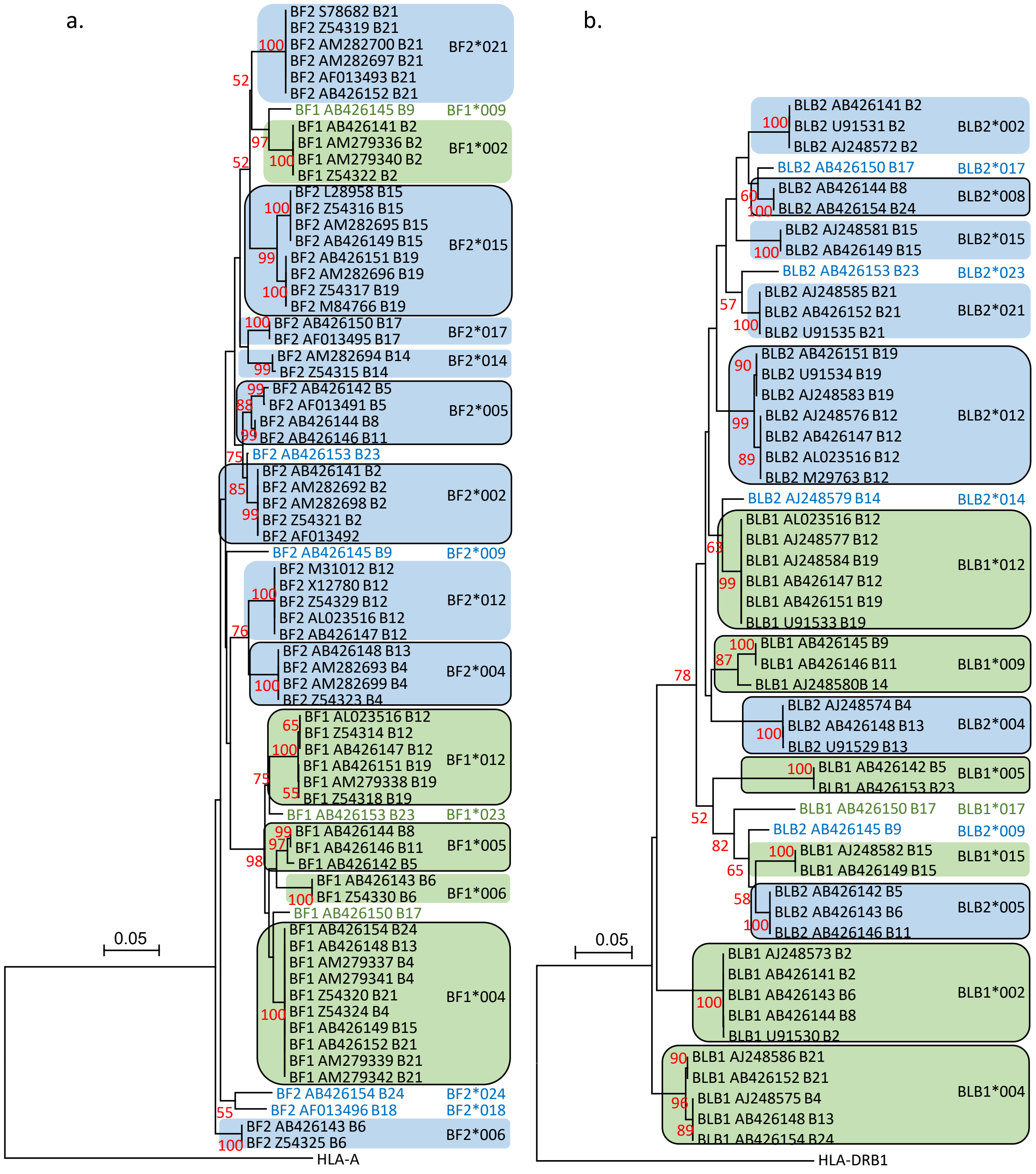

Online Resource 3. Phylogenetic trees for nucleotide sequences of exons encoding MHC peptide-binding domains from standard haplotypes. a. exons 2 and 3 of BF sequences (with the first 20 nucleotides of exon 2 and the last 23 nucleotides of exon 3 removed, corresponding to primers and other reasons for different lengths of sequence); b. exon 2 of BLB sequences (with the first 6-8 nucleotides removed, corresponding to primers and other reasons for different lengths of sequence). Genetic distances are indicated with bars; red numbers are bootstrap values (percentages) for those nodes that reach significance from 500 replications; names at the tips are the gene name, followed by the GenBank accession number, followed by the haplotype. Allele groups for BF1 and BLB1 (or BF2 and BLB2) are named, either in green (or blue) for single sequences or in black surrounded by green (or blue) background for clades with more than one sequence; the coloured background for clades with sequences from more than one haplotype are surrounded by a black line. Human sequences were used as outgroups; sequences for standard haplotypes were taken from the GenBank accession numbers in Online Resource 2; all other details are as in the legend to Online Resource 1.

a.

BF1	B2	B4	B5	B6	B8	B9	B11	B12	B13	B15	B17	B19	B21	B23	B24
B2	ID														
B4	53	ID													
B5	50	18	ID												
B6	46	26	24	ID											
B8	46	16	4	20	ID										
B9	20	54	48	52	52	ID									
B11	46	16	4	20	0	52	ID								
B12	57	25	28	36	32	51	32	ID							
B13	53	0	18	26	16	54	16	25	ID						
B15	53	0	18	26	16	54	16	25	0	ID					
B17	53	12	17	25	15	52	15	29	12	12	ID				
B19	57	24	27	35	31	51	31	1	24	24	28	ID			
B21	53	0	18	26	16	54	16	25	0	0	12	24	ID		
B23	57	16	24	34	26	47	26	21	16	16	22	20	16	ID	
B24	53	0	18	26	16	54	16	25	0	0	12	24	0	16	ID

b.

BF2	B2	B4	B5	B6	B8	B9	B11	B12	B13	B14	B15	B17	B18	B19	B21	B23	B24
B2	ID																
B4	39	ID															
B5	18	40	ID														
B6	39	41	40	ID													
B8	12	39	9	37	ID												
B9	34	46	35	35	36	ID											
B11	12	39	9	37	0	36	ID										
B12	19	24	29	38	27	36	27	ID									
B13	39	0	40	41	39	46	39	24	ID								
B14	28	41	28	37	26	36	26	33	41	ID							
B15	36	53	38	39	37	35	37	52	53	25	ID						
B17	23	43	21	39	24	35	24	33	43	20	33	ID					
B18	36	35	34	32	37	38	37	35	35	25	41	31	ID				
B19	33	48	33	36	34	35	34	47	48	28	10	33	42	ID			
B21	30	43	30	41	28	40	28	45	43	34	35	31	44	27	ID		
B23	5	38	14	34	7	34	7	22	38	23	34	18	32	31	29	ID	
B24	35	44	34	34	39	40	39	34	44	41	52	34	29	51	49	34	ID

c.

BF2/BF1	B2	B4	B5	B6	B8	B9	B11	B12	B13	B15	B17	B19	B21	B23	B24
B2	38	40	34	46	38	39	38	44	40	40	38	44	40	40	40
B4	47	40	53	51	49	48	49	49	40	40	46	49	40	44	40
B5	38	47	40	45	44	34	44	43	47	47	43	43	47	43	47
B6	35	41	43	35	39	37	39	50	41	41	42	50	41	43	41
B8	37	38	31	45	35	31	35	38	38	38	34	38	38	34	38
B9	42	42	41	49	43	40	43	46	42	42	49	46	42	45	42
B11	37	38	31	45	35	31	35	38	38	38	34	38	38	34	38
B12	48	43	43	47	47	48	47	45	43	43	49	45	43	41	43
B13	47	40	53	51	49	48	49	49	40	40	46	49	40	44	40
B14	46	49	42	42	46	41	46	47	49	49	40	47	49	51	49
B15	35	54	48	50	50	36	50	58	54	54	45	58	54	58	54
B17	45	46	40	38	44	39	44	50	46	46	40	50	46	45	46
B18	47	49	52	45	50	48	50	50	49	49	46	50	49	49	49
B19	35	55	47	49	51	34	51	58	55	55	48	58	55	56	55
B21	35	53	48	47	52	28	52	48	53	53	51	48	53	51	53
B23	36	35	29	41	33	34	33	39	35	35	33	39	35	35	35
B24	44	49	51	44	47	50	47	56	49	49	55	55	49	51	49

d.

BLB1	B2	B4	B5	B6	B8	B9	B11	B12	B13	B14	B15	B17	B19	B21	B23	B24
B2	ID															
B4	26	ID														
B5	45	46	ID													
B6	0	26	45	ID												
B8	0	26	45	0	ID											
B9	37	31	35	37	37	ID										
B11	37	31	35	37	37	0	ID									
B12	33	25	35	33	33	19	19	ID								
B13	26	0	46	26	26	31	31	25	ID							
B14	30	31	31	30	30	7	7	18	31	ID						
B15	40	45	38	40	40	28	28	28	45	26	ID					
B17	38	46	33	38	38	34	34	32	46	31	23	ID				
B19	33	25	35	33	33	19	19	0	25	18	28	32	ID			
B21	24	2	46	24	24	33	33	25	2	33	45	44	25	ID		
B23	45	46	0	45	45	35	35	35	46	31	38	33	35	46	ID	
B24	26	0	46	26	26	31	31	25	0	31	45	46	25	2	46	ID

e.

BLB2	B2	B4	B5	B6	B8	B9	B11	B12	B13	B14	B15	B17	B19	B21	B23	B24
B2	ID															
B4	31	ID														
B5	34	36	ID													
B6	34	36	0	ID												
B8	12	31	32	32	ID											
B9	36	36	10	10	33	ID										
B11	34	36	0	0	32	10	ID									
B12	16	29	30	30	21	28	30	ID								
B13	31	0	36	36	31	36	36	29	ID							
B14	26	21	24	24	25	20	24	19	21	ID						
B15	21	35	34	34	14	35	34	26	35	25	ID					
B17	17	29	29	29	7	29	29	22	29	23	20	ID				
B19	16	31	30	30	19	28	30	2	31	21	24	20	ID			
B21	21	24	28	28	13	25	28	20	24	20	19	12	20	ID		
B23	29	28	33	33	24	30	33	24	28	25	24	19	24	12	ID	
B24	12	31	32	32	0	33	32	21	31	25	14	7	19	13	24	ID

f.

BLB2/BLB1	B2	B4	B5	B6	B8	B9	B11	B12	B13	B14	B15	B17	B19	B21	B23	B24
B2	41	38	44	41	41	33	33	24	38	34	43	38	24	36	44	38
B4	39	42	40	39	39	21	21	25	42	28	38	40	25	40	40	42
B5	39	39	37	39	39	30	30	25	39	25	12	18	25	37	37	39
B6	39	39	37	39	39	30	30	25	39	25	12	18	25	37	37	39
B8	43	34	41	43	43	28	28	20	34	29	41	38	20	32	41	34
B9	35	37	41	35	35	32	32	25	37	27	14	24	25	35	41	37
B11	39	39	37	39	39	30	30	25	39	25	12	18	25	37	37	39
B12	35	30	42	35	35	26	26	18	30	26	38	34	18	28	42	30
B13	39	42	40	39	39	21	21	25	42	28	38	40	25	40	40	42
B14	30	27	36	30	30	18	18	9	27	17	29	29	9	27	36	27
B15	35	32	35	35	35	23	23	22	32	24	37	37	22	32	35	32
B17	38	33	36	38	38	27	27	21	33	23	39	36	21	31	36	33
B19	37	28	40	37	37	26	26	16	28	26	38	35	16	26	40	28
B21	34	34	34	34	34	21	21	20	34	18	33	34	20	32	34	34
B23	35	33	32	35	35	20	20	23	33	16	33	36	23	35	32	33
B24	43	34	41	43	43	28	28	20	34	29	41	38	20	32	41	34

Online resource 4. Distance matrices for nucleotide sequences of exons encoding MHC peptide-binding domains from standard haplotypes. Exons 2 and 3 of a. BF1 versus BF1 alleles, b. BF2 versus BF2 alleles, c. BF1 versus BF2 alleles; d. BLB1 versus BLB1 alleles, e. BLB2 versus BLB2 alleles, f. BLB1 versus BLB2 alleles. Sequences for standard haplotypes were taken from the GenBank accession numbers in Online Resource 2. Alignments were performed using MAFFT on-line [Katoh K, Misawa K, Kuma K, Miyata T (2002) MAFFT: a novel method for rapid multiple sequence alignment based on fast Fourier transform. Nucleic Acids Res 30:3059-3066; https://mafft.cbrc.jp/alignment/server/] and the results were pasted into Bioedit [Hall TA (1999) BioEdit: a user-friendly biological sequence alignment editor and analysis program for Windows 95/98/NT. Nucl. Acids. Symp. Ser. 41:95-98; https://softfamous.com/bioedit/] on a desktop computer; the command "Sequence difference count Matrix" under "Alignment" was used to generate the distance matrix, which was pasted into Microsoft Excel and then Powerpoint for producing the final figure. Highlights indicate amino acid differences for BF (or BLB) from Fig. 3 (for comparison to nucleotide differences in this figure): green, none; blue, 1 to 4 (1 or 2); yellow, 5 to 8 (3 or 4); ID, comparison between the same sequence.

a.

BF1	B2	B4	B5	B6	B8	B9	B11	B12	B13	B15	B17	B19	B21	B23	B24
B2	ID														
B4	47	ID													
B5	43	17	ID												
B6	40	18	16	ID											
B8	39	15	4	12	ID										
B9	23	54	46	51	50	ID									
B11	39	15	4	12	0	50	ID								
B12	41	30	28	33	32	44	32	ID							
B13	47	0	17	18	15	54	15	30	ID						
B15	45	2	17	18	15	54	15	30	2	ID					
B17	47	11	19	20	17	53	17	33	11	9	ID				
B19	41	29	27	32	31	44	31	1	29	29	32	ID			
B21	45	2	17	18	15	54	15	30	2	0	9	29	ID		
B23	44	22	27	31	28	43	28	20	22	22	26	19	22	ID	
B24	47	2	19	20	17	56	17	32	2	2	11	31	2	24	ID

d.

BLB1	B2	B5	B6	B8	B9	B11	B12	B13	B15	B17	B19	B21	B23	B24
B2	ID													
B5	23	ID												
B6	0	23	ID											
B8	0	23	0	ID										
B9	22	22	22	22	ID									
B11	22	22	22	22	0	ID								
B12	18	20	18	18	16	16	ID							
B13	17	25	17	17	20	20	17	ID						
B15	24	22	24	24	20	20	20	26	ID					
B17	23	19	23	23	20	20	21	27	16	ID				
B19	18	20	18	18	16	16	0	17	20	21	ID			
B21	16	24	16	16	21	21	16	1	25	26	16	ID		
B23	35	12	35	35	34	34	32	37	34	31	32	36	ID	
B24	17	25	17	17	20	20	17	0	26	27	17	1	37	ID

b.

BF2	B2	B4	B5	B6	B8	B9	B11	B12	B13	B14	B15	B17	B18	B19	B21	B23	B24
B2	ID																
B4	27	ID															
B5	17	32	ID														
B6	33	31	33	ID													
B8	15	31	5	33	ID												
B9	31	36	30	37	31	ID											
B11	15	31	5	33	0	31	ID										
B12	11	18	24	31	23	32	23	ID									
B13	27	0	32	31	31	36	31	18	ID								
B14	24	31	26	34	25	32	25	27	31	ID							
B15	27	35	31	33	30	33	30	35	35	21	ID						
B17	21	34	21	35	23	29	23	28	34	23	28	ID					
B18	44	44	47	47	48	50	48	44	44	43	48	45	ID				
B19	24	31	26	28	27	32	27	30	31	22	10	26	49	ID			
B21	24	31	23	35	24	32	24	32	31	28	28	26	52	22	ID		
B23	5	26	13	29	10	31	10	15	26	20	25	16	40	22	24	ID	
B24	29	33	32	29	35	38	35	30	33	36	39	31	46	38	37	29	ID

e.

BLB2	B2	B5	B6	B8	B9	B11	B12	B13	B15	B17	B19	B21	B23	B24
B2	ID													
B5	23	ID												
B6	23	0	ID											
B8	8	22	22	ID										
B9	22	11	11	20	ID									
B11	23	0	0	22	11	ID								
B12	11	20	20	14	17	20	ID							
B13	21	20	20	20	22	20	20	ID						
B15	15	22	22	11	23	22	18	20	ID					
B17	13	22	22	7	20	22	17	21	17	ID				
B19	11	20	20	12	17	20	2	20	16	15	ID			
B21	15	20	20	10	18	20	14	18	14	13	14	ID		
B23	18	21	21	15	19	21	15	18	17	14	15	9	ID	
B24	8	22	22	0	20	22	14	20	11	7	12	10	15	ID

c.

BF2/BF1	B2	B4	B5	B6	B8	B9	B11	B12	B13	B15	B17	B19	B21	B23	B24
B2	36	41	37	43	41	42	41	31	41	39	40	31	39	34	41
B4	33	39	47	42	43	45	43	34	39	37	42	34	37	35	39
B5	36	43	39	42	43	41	43	30	43	41	42	30	41	36	43
B6	34	43	44	36	40	42	40	37	43	41	42	37	41	38	43
B8	35	38	34	43	38	39	38	29	38	36	37	29	36	31	38
B9	39	43	40	45	42	42	42	33	43	41	46	33	41	37	43
B11	35	38	34	43	38	39	38	29	38	36	37	29	36	31	38
B12	40	44	42	45	46	46	46	32	44	42	46	32	42	35	44
B13	33	39	47	42	43	45	43	34	39	37	42	34	37	35	39
B14	40	44	39	42	43	44	43	28	44	42	41	28	42	38	44
B15	34	43	41	42	43	39	43	33	43	43	42	33	43	38	45
B17	41	43	39	41	43	42	43	32	43	41	38	32	41	33	43
B18	57	60	62	57	60	62	60	51	60	60	62	51	60	56	62
B19	33	43	40	41	44	36	44	32	43	43	41	32	43	38	45
B21	31	46	40	40	44	35	44	33	46	44	44	33	44	39	46
B23	34	36	32	39	36	39	36	26	36	34	35	26	34	29	36
B24	41	44	46	39	42	53	42	38	44	42	46	38	42	39	44

f.

BLB2/BLB1	B2	B5	B6	B8	B9	B11	B12	B13	B15	B17	B19	B21	B23	B24
B2	24	25	24	24	22	22	15	21	25	24	15	20	37	21
B5	26	21	26	26	18	18	18	24	9	12	18	23	33	24
B6	26	21	26	26	18	18	18	24	9	12	18	23	33	24
B8	24	23	24	24	20	20	13	20	25	25	13	19	35	20
B9	22	23	22	22	20	20	17	21	12	19	17	20	35	21
B11	26	21	26	26	18	18	18	24	9	12	18	23	33	24
B12	22	23	22	22	17	17	13	18	24	22	13	17	35	18
B13	22	23	22	22	14	14	14	24	24	21	14	23	35	24
B15	19	22	19	19	18	18	15	21	25	24	15	20	34	21
B17	24	23	24	24	22	22	16	21	26	26	16	20	34	21
B19	23	21	23	23	17	17	11	16	24	22	11	15	33	16
B21	21	20	21	21	17	17	15	22	22	24	15	21	32	22
B23	20	20	20	20	14	14	16	18	22	24	16	19	32	18
B24	24	23	24	24	20	20	13	20	25	25	13	19	35	20

Online Resource 5. Distance matrices for amino acids of whole coding sequences (CDS) from standard haplotypes. a. BF1 versus BF1 alleles, b. BF2 versus BF2 alleles, c. BF1 versus BF2 alleles; d. BLB1 versus BLB1 alleles, e. BLB2 versus BLB2 alleles, f. BLB1 versus BLB2 alleles. All details as in legend to Online Resource 4, except that the names of sequences with indels are highlighted: grey, insertion (one amino acid in BF1, two amino acids in BF2); pink, deletion (five amino acids in BF1, 11 amino acids in BF2); orange, a combination of a one nucleotide deletion and a five nucleotide truncation leading to a frameshift at amino acid 247 in the transmembrane region (BLB1 from the B23 haplotype).

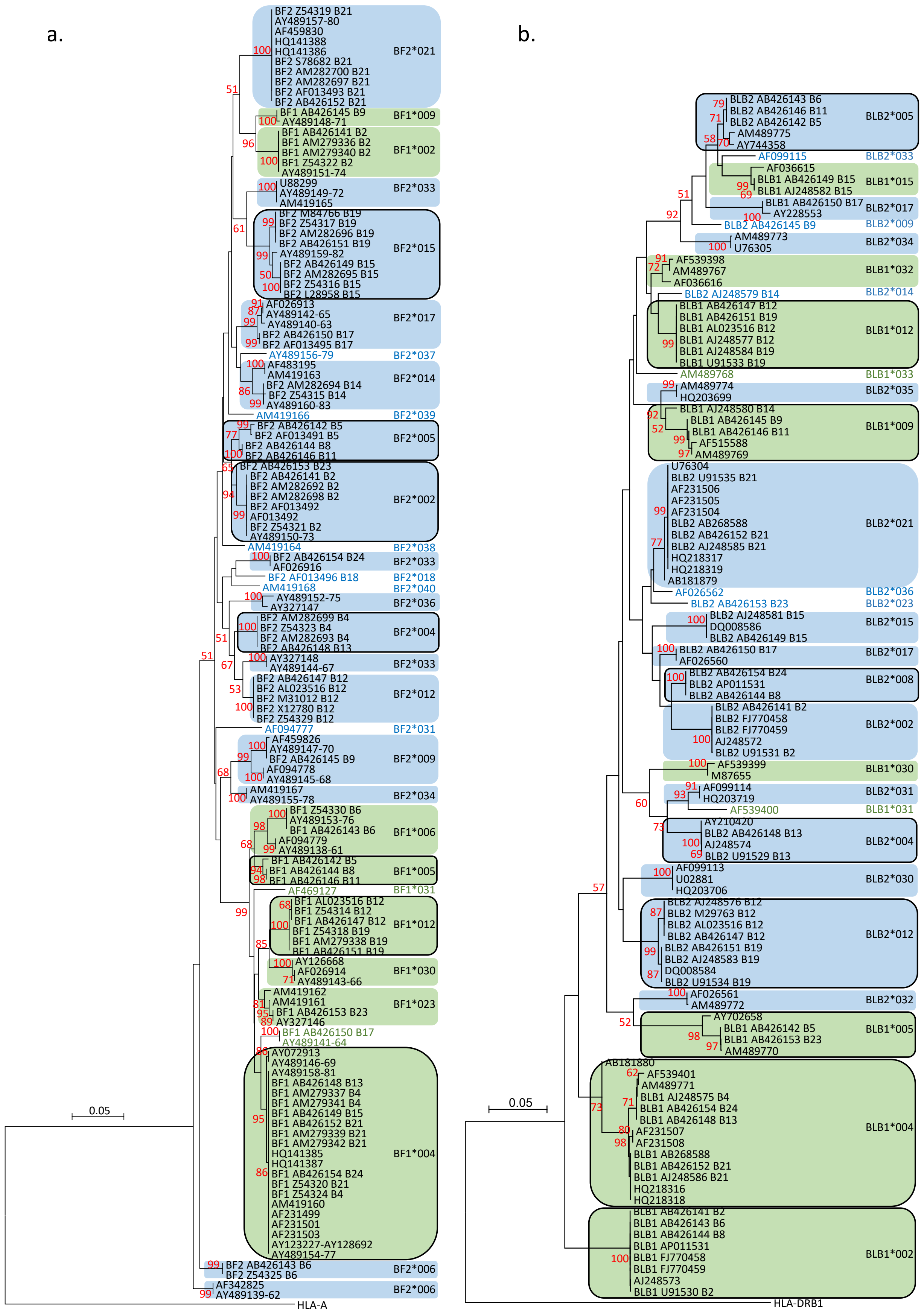

Online Resource 6. Phylogenetic trees for nucleotide sequences of exons encoding MHC peptide-binding domains from standard haplotypes and from sequences in the scientific literature. a. exons 2 and 3 of BF sequences (with the first 20 nucleotides of exon 2 and the last 23 nucleotides of exon 3 removed, corresponding to primers and other reasons for different lengths of sequence); b. exon 2 of BLB sequences (with the first 6-8 nucleotides removed, corresponding to primers and other reasons for different lengths of sequence). Sequences for standard haplotypes and from the scientific literature were taken from the GenBank accession numbers in Online Resource 2; all other details are as in the legends to Online Resources 1 and 3.
